# Densities and numbers of calbindin and parvalbumin positive neurons across the rat and mouse brain

**DOI:** 10.1016/j.isci.2020.101906

**Published:** 2020-12-08

**Authors:** Ingvild E. Bjerke, Sharon C. Yates, Arthur Laja, Menno P. Witter, Maja A. Puchades, Jan G. Bjaalie, Trygve B. Leergaard

**Affiliations:** 1 Department of Molecular Medicine, Institute of Basic Medical Sciences, University of Oslo, Oslo, Norway; 2 Kavli Institute for Systems Neuroscience, Norwegian University of Science and Technology, Trondheim, Norway

**Keywords:** Histology, Imaging Anatomy, Optical Imaging, Molecular Neuroscience, Cellular Neuroscience

## Abstract

The calcium-binding proteins parvalbumin and calbindin are expressed in neuronal populations regulating brain networks involved in spatial navigation, memory processes, and social interactions. Information about the numbers of these neurons across brain regions is required to understand their functional roles but is scarcely available. Employing semi-automated image analysis, we performed brain-wide analysis of immunohistochemically stained parvalbumin and calbindin sections and show that these neurons distribute in complementary patterns across the mouse brain. Parvalbumin neurons dominate in areas related to sensorimotor processing and navigation, whereas calbindin neurons prevail in regions reflecting behavioral states. We also find that parvalbumin neurons distribute according to similar principles in the hippocampal region of the rat and mouse brain. We validated our results against manual counts and evaluated variability of results among researchers. Comparison of our results to previous reports showed that neuron numbers vary, whereas patterns of relative densities and numbers are consistent.

## Highlights

• Brain-wide, semi-automatic quantification of parvalbumin and calbindin neurons
• Largely complementary distribution of calbindin and parvalbumin neurons in mice
• Comparison with several previous studies shows variable numbers but similar trends
• Similar distribution of parvalbumin neurons in the rat and mouse hippocampal region

## Graphical Abstract

**Figure:**
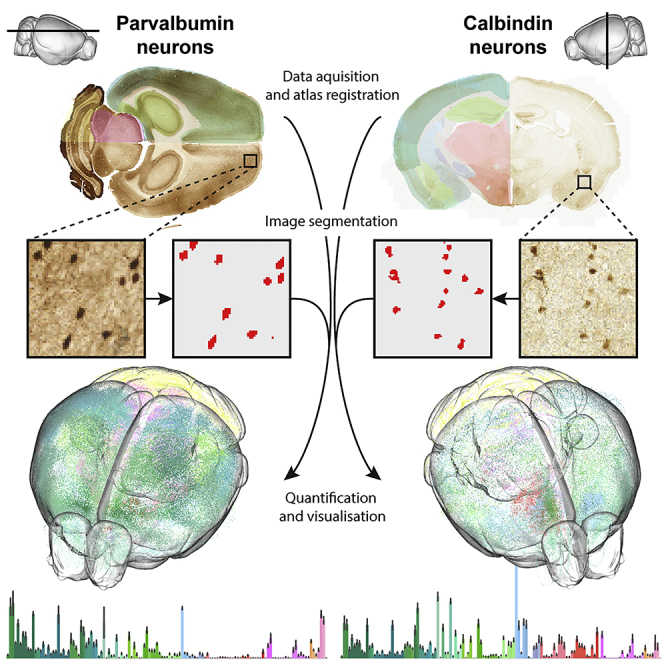

## Introduction

Transient increases in intracellular calcium concentrations play a critical role in the regulation of neuronal excitability, neurotransmitter release, and synaptic plasticity (Berridge, 1998). The spatial and temporal dynamics of such calcium signals can be modulated by calcium-binding proteins, which are widely expressed in the nervous system (Schwaller, 2010). Two such proteins, parvalbumin and calbindin, are expressed in largely non-overlapping groups of neurons that show fast-spiking and bursting electrophysiological phenotypes, respectively (Markram et al., 2004).

Parvalbumin is expressed in a group of interneurons characterized by fast responses and effective inhibition of surrounding principal neurons (Hu et al., 2014). The role of parvalbumin neurons in fine-tuning networks of principal neurons has been widely investigated in deep layers of somatosensory and visual cortices (Atallah et al., 2012; Runyan et al., 2010; Yu et al., 2016, 2019a). In association with cortices, parvalbumin neurons are less prominent in deeper layers, and interestingly, in parahippocampal domains they are primarily seen in superficial layers (Boccara et al., 2015). A well-studied example is the parvalbumin neuron in the medial entorhinal cortex, known for its characteristic grid cells, which have multiple firing fields making up a triangular array across the entire environment available to an animal (Hafting et al., 2005). Parvalbumin interneurons are key modulators of these cells, particularly in layer II networks, where principal neurons communicate through parvalbumin interneurons (Couey et al., 2013; Miao et al., 2017). Similar principles of inhibitory connectivity has been shown in the lateral entorhinal cortex (Nilssen et al., 2018), where principal cells are tuned to the past and present positions of objects (Tsao et al., 2013) and groups of cells are involved in representing sequences of event (Tsao et al., 2018). Beyond the role of parvalbumin neurons in parahippocampal circuits, the importance of these interneurons across the brain is supported by their dysfunction in several neuropsychiatric and developmental disorders (Ferguson and Gao, 2018; for review, see Marín, 2012), including autism spectrum disorders (Gogolla et al., 2009), Tourette syndrome (Kalanithi et al., 2005) and schizophrenia (Gonzalez-Burgos and Lewis, 2012; Hashimoto et al., 2003).

Calbindin-D28k is expressed in populations of excitatory and inhibitory neurons (Jinno and Kosaka, 2006; Szabadics et al., 2010). Other calbindin proteins include calbindin-D9k, primarily expressed in epithelial cells, and calretinin, which is expressed in neuronal cells and to a degree co-localizes with calbindin-D28k (Lu et al., 2009; Rogers and Résibois, 1992). In this study, we focus on neurons expressing calbindin-D28k, in the following referred to as calbindin neurons. In the neocortex, calbindin is typically associated with interneurons (Ascoli et al., 2008; Markram et al., 2004), but this protein is also found to be expressed in pyramidal neurons, e.g. in the medial entorhinal cortex (Ray et al., 2014) and CA1 (Merino-Serrais et al., 2020). Calbindin-positive neurons thus probably represent both interneurons and principal neurons, perhaps depending on the area in question, and the functional roles of calbindin neurons have been less well characterized than those of parvalbumin interneurons. However, recent evidence shows that selective knockdown of calbindin neurons in the CA1 and dentate gyrus regions of the hippocampus can reduce long-term potentiation, pointing to a role for these neurons in memory function (Li et al., 2017). Calbindin neurons have also been implicated in fear memory and social behavior (Harris et al., 2016) and have been hypothesized to have a neuroprotective role (Sun et al., 2011).

Given the well-known and proposed roles of parvalbumin and calbindin neurons in neuronal networks, quantitative information representing their number and distribution in the brain is of broad interest to neuroscientists. Such data are needed to constrain computational models, to measure group differences in intervention-based studies, and to draw conclusions about structure-function relationships. Several studies have quantified neurons expressing calcium-binding proteins in one or a few brain regions (see, e.g. Andsberg et al., 2001; Pitts et al., 2013; Schmid et al., 2013; Yalcin-Cakmakli et al., 2018). Studies on a larger scale have typically been qualitative or semi-quantitative (Arai et al., 1994; Frantz and Tobin, 1994), whereas one study has reported brain-wide quantitative data about parvalbumin neurons in Cre reporter mice (Kim et al., 2017). Others have focused on gathering measurements from the literature and calculating values for parameters that have yet to be tested experimentally (Bezaire and Soltesz, 2013). However, there is growing awareness that numbers reported in the literature are prone to substantial variability across publications (Bjerke et al., 2020a; Keller et al., 2018). Quantitative studies of parvalbumin and calbindin neurons acquired across the brain are needed to elucidate their relative numbers and distributions within and across regions and species. Also, replication and validation of quantitative studies will be essential to converge on realistic estimates of the number of various cell types.

Computational methods for automated segmentation, localization, and quantification of cells have successfully been applied to three-dimensional volumetric datasets to generate region- or brain-wide estimates of cell numbers in mice (Kim et al., 2017; Murakami et al., 2018; Silvestri et al., 2015; Zhang et al., 2017). These efforts have relied on advanced volumetric imaging techniques, genetically modified animals expressing fluorescent signals in cells of interest, and custom codes for analysis. Immunohistochemical techniques continue to serve important purposes for characterizing cell populations based on protein expression (which may only be a subset of those expressing the gene for the protein). To achieve efficient quantification of immunohistochemically labeled cells in sectioned material, we used the QUINT workflow (Yates et al., 2019), which combines three open-access tools, QuickNII (Puchades et al., 2019), ilastik (Berg et al., 2019), and Nutil (Groeneboom et al., 2020). This workflow achieves quantification of segmented objects in atlas-defined regions of interest, using customized brain atlas maps, section coordinates, and machine-learning-based segmentation of the labeled objects.

We here ask how the numbers and spatial distributions of neurons expressing calcium-binding proteins vary across brain regions and possibly relate to functional or topographical patterns of organization. We quantify two largely distinct cell types identified by the calcium-binding proteins parvalbumin and calbindin in the mouse brain using the QUINT workflow and compare these with previous reports. We further quantify parvalbumin neurons in the entire rat brain and perform a detailed comparison of parvalbumin neuron numbers in the mouse and rat hippocampal regions. We validate the resulting numbers with manual counts in selected areas and assess the reliability of segmentation results between researchers. All the raw and derived datasets presented here are shared through the EBRAINS Knowledge Graph to facilitate further analysis and re-use.

## Results

We used the QuickNII-ilastik-Nutil (QUINT) workflow to quantify parvalbumin neurons in the mouse and rat brain, and calbindin neurons in the mouse brain, corrected the resulting numbers with Abercrombie's formula, and extrapolated corrected numbers to represent whole regions and volumetric densities. All data were anatomically located using the Allen Mouse Common Coordinate Framework, version 3 of the template, 2017 edition of the delineations (Wang et al., 2020; hereafter referred to as CCFv3-2017) and the Waxholm Space atlas of the Sprague-Dawley rat brain, version 1.01 of the template and version 2 of the delineations (Papp et al., 2014; Kjonigsen et al., 2015; hereafter referred to as WHSv2). Details about all procedures are provided in the Transparent methods section.

**Figure 1:**
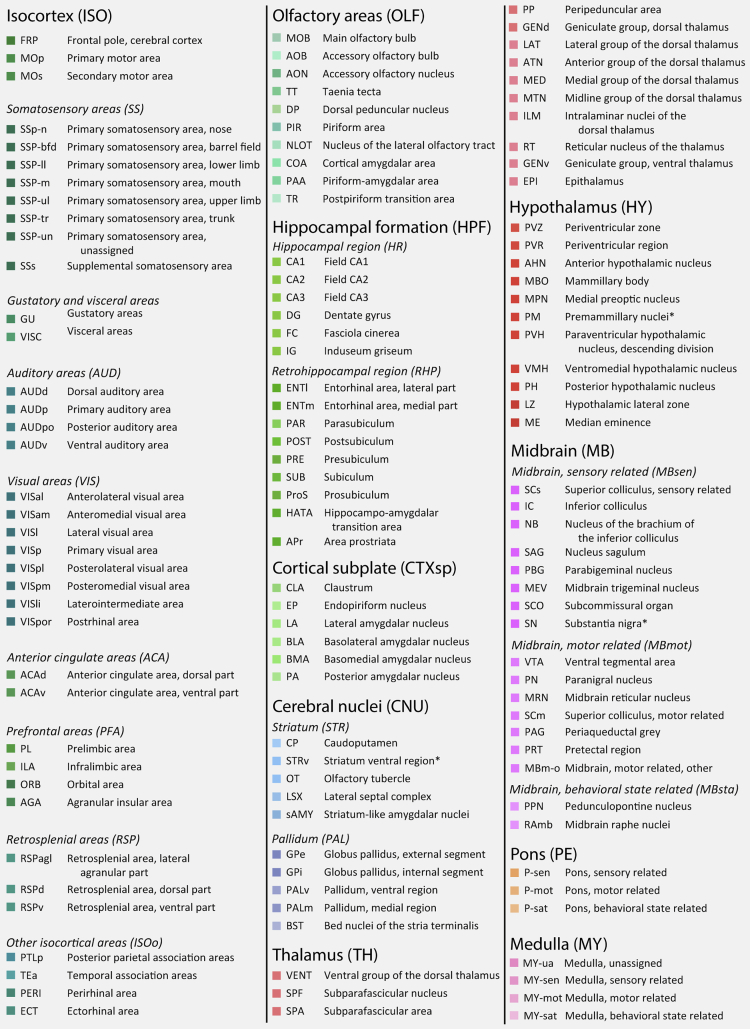
Custom regions of interest used for analysis of mouse brain data Color codes and abbreviations for the custom regions used in Nutil Quantifier. These are consistent with the CCFv3-2017 nomenclature, except the three marked with an asterisk. Main titles correspond to high-level regions, whereas italic subtitles correspond to finer regions.

Below, we first present the quantitative data on the densities of parvalbumin and calbindin neurons in the mouse brain (n = 4 and 5, respectively), as density estimates are readily compared across regions of variable size. We go on to compare the total numbers of these cell types across the brain and analyze their relative numbers in each brain region. In addition to the text and figures presented here, all numbers and density estimates are listed in Table S1. We then compare the total number, density, and distribution of parvalbumin neurons in the mouse (n = 4) and rat (n = 4) hippocampal regions. Lastly, we compare our findings to numbers reported in the literature and assess the validity and reliability of QUINT results. All numbers reported are given as mean ± SEM; total number estimates are bilateral, whereas densities are given per mm^3^. The nomenclature used here for mouse anatomical regions follows the CCFv3-2017 hierarchy (except in the cross-species comparison, where WHSv2 terms are used for both species). Some of the overarching terms from the CCFv3-2017 may not be commonly used by researchers; however, these are all listed and explained in Figure 1.

### Parvalbumin neuron densities across the mouse brain

**Figure 2:**
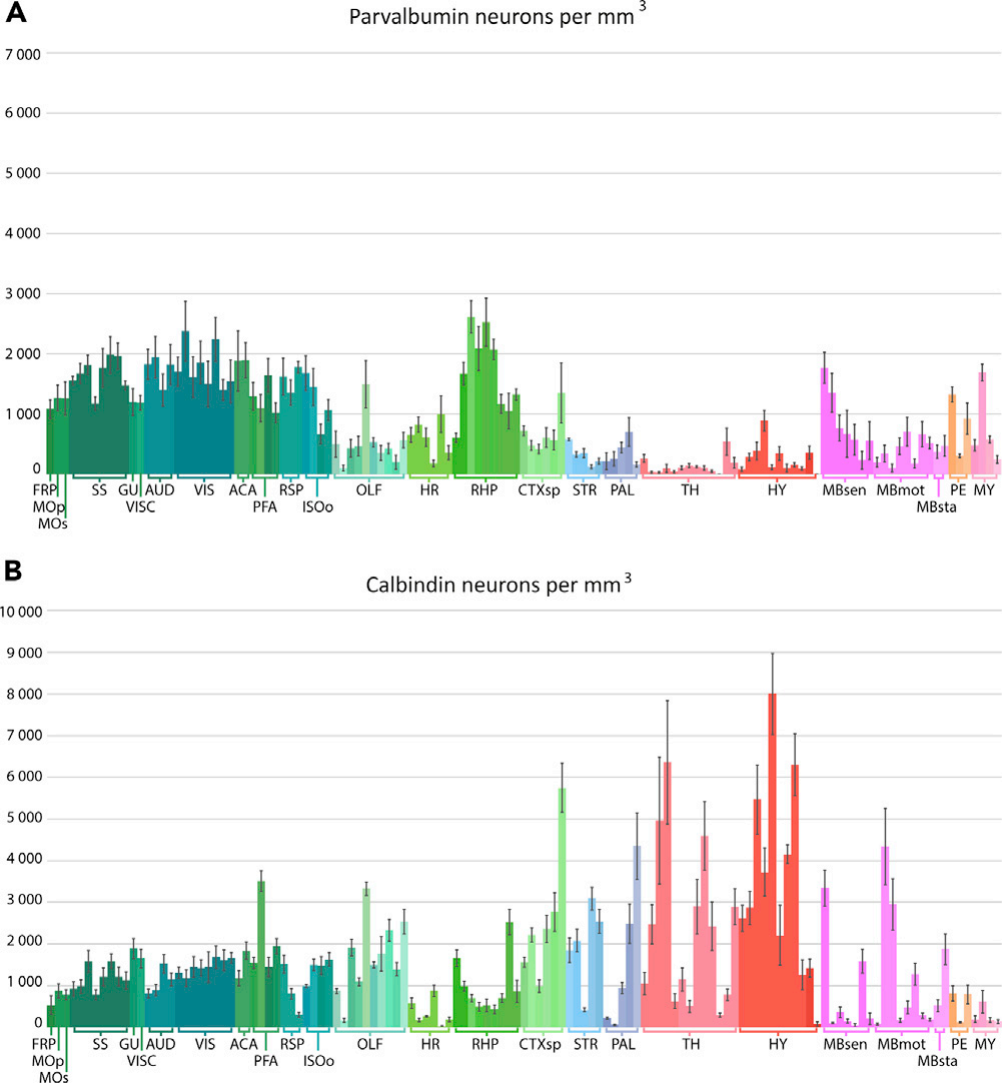
Parvalbumin and calbindin neuron densities across mouse brain regions Bar graph showing the mean density per mm^3^ of parvalbumin (n = 4; (A)) and calbindin (n = 5, (B)) neurons across the brain. Error bars indicate SEM. Groups of brain areas are indicated along the x axis. Bars are placed from left to right in the same order as abbreviations are listed and explained in Figure 1. See Table S1 for an overview of all the density estimates.

Parvalbumin neurons were most densely packed in isocortical areas and in the retrohippocampal region. Olfactory areas and areas of the hippocampal region, striatum, pallidum, and cortical subplate generally showed moderate densities, with some olfactory and amygdalar areas having high densities (Figure 2A; for details see below). Low densities were seen in the thalamus and hypothalamus, although some areas stood out with moderate amounts of parvalbumin neurons. Midbrain, pontine, and medullary regions generally showed low and moderate parvalbumin neuron densities. The density estimates for parvalbumin neurons in all gray matter regions of the mouse brain are summarized in Figure 2A, and all total number and density estimates for all mouse brain regions are included in the derived dataset.

*Isocortex.* Relatively high densities were seen across most isocortical areas. Auditory, visual, and somatosensory areas generally showed slightly higher densities than gustatory, visceral, and prefrontal areas. Among isocortical areas, the highest parvalbumin neuron density was seen in the anteromedial visual area (2376 ± 496), whereas the most sparse distribution was seen in the perirhinal area (666 ± 165).

*Olfactory areas.* The dorsal peduncular area showed the highest density of the olfactory areas (1493 ± 394), in contrast to a very low density in the accessory olfactory bulb (100 ± 48). Other olfactory areas showed a moderate density.

*Hippocampal formation.* The hippocampal region showed moderate densities of parvalbumin neurons. In the retrohippocampal regions, densities were generally high, with the parasubiculum showing the highest parvalbumin neuron density of all mouse brain regions (2614 ± 269). A detailed account of parvalbumin neuron densities across the hippocampal formation is given below.

*Cortical subplate.* Moderate parvalbumin neuron densities were seen across claustrum, entopeduncular, and amygdalar regions. The posterior amygdalar nucleus stood out among the cortical subplate regions with a relatively high density (1349 ± 499).

*Cerebral nuclei.* In the striatum, the dorsal (caudoputamen) part showed a higher density of parvalbumin neurons (577 ± 20) than the ventral (nucleus accumbens and fundus of striatum) region (327 ± 43). Among regions of the pallidum, the medial region showed the highest density (701 ± 238).

*Thalamus and hypothalamus.* The thalamic regions generally had low densities of parvalbumin neurons, except for the reticular nucleus (but this region was oversaturated with staining, and numbers are therefore not reported). The geniculate group of the ventral thalamus also showed a relatively high density (539 ± 225). The mammillary body showed the highest density among the hypothalamic regions (888 ± 172).

*Midbrain.* In the superior colliculus, a higher density was seen in the superficial (sensory related) part (1768 ± 257) than in deeper (motor related) layers (704 ± 238). The inferior colliculus (1352 ± 320) and the pretectal region (663 ± 209) also showed relatively high densities compared with other midbrain regions.

*Pons and medulla.* Pontine and medullary regions were grouped into quite broad categories for the current analysis, but sensory related parts showed higher densities than the motor and behavioral state related ones (pons, sensory related: 1324 ± 126; medulla, sensory related: 1690 ± 140). Parvalbumin staining was seen across cerebellar layers, with Purkinje cell bodies darkly stained, but the staining was oversaturated, preventing extraction of cell numbers and densities.

### Calbindin neuron densities across the mouse brain

Calbindin neurons were mapped and quantified throughout the mouse brain. In cortical areas, lightly stained but densely packed cells were typically seen in layer II, whereas deeper layers had a more scattered distribution of strongly stained cells. Density estimates for calbindin neurons in all regions are shown in Figure 2B.

*Isocortex.* Of the isocortical areas, the infralimbic area showed an especially high density (3506 ± 249), whereas the frontal pole showed a much more sparser distribution of calbindin neurons (532 ± 219). Within the primary somatosensory area, the trunk region showed the highest density (1582 ± 275), whereas the mouth region showed the lowest (782 ± 120). Densities were similar among the auditory and visual areas. Among retrosplenial areas, distinct differences were seen, with the ventral part having a lower density (306 ± 55) than the dorsal part (803 ± 130). The lateral agranular part of the retrosplenial cortex showed a higher density than both of these (1512 ± 218).

*Olfactory areas.* Olfactory areas showed a relatively high density of calbindin neurons, with an especially high density seen in the dorsal peduncular area (3324 ± 156). The accessory olfactory bulb, however, showed a very sparse distribution of calbindin neurons (164 ± 48).

*Hippocampal formation.* The hippocampal areas showed quite low calbindin neuron densities, with the dentate gyrus showing the highest density (876 ± 136). Retrohippocampal regions also generally showed low densities, except for the entorhinal area, lateral part with a moderate density (1659 ± 197), and the hippocampo-amygdalar transition area, which showed a relatively high calbindin neuron density (2519 ± 303).

*Cortical subplate.* The claustrum and entopeduncular nucleus showed relatively high calbindin neuron densities, as did all amygdalar areas, except for the lateral amygdalar nucleus (986 ± 154). The density in the posterior amygdalar nucleus was considerably higher than all other subregions (5745 ± 589) of the cortical subplate.

*Cerebral nuclei.* Striatal regions generally showed relatively high densities of calbindin neurons, except for the olfactory tubercle that was very sparsely populated (418 ± 48). The caudoputamen had the highest total number of calbindin neurons of all brain regions (48064 ± 7665), but due to the large size of this region, the density was not considerably higher than other regions (1847 ± 295). However, we note that both the caudoputamen and striatum ventral region contained a large population of lightly stained cells, which as mentioned in the methods were not completely represented with our classifier. In the pallidum, quite low density was seen in the globus pallidus, both external (223 ± 24) and internal (57 ± 16) segments, whereas the ventral and medial regions of the pallidum showed a higher density, especially the latter (2492 ± 471).

*Thalamus and hypothalamus.* In the thalamus, especially high densities were seen in the subparafascicular nucleus (2474 ± 474) and the subparafascicular area (4964 ± 1526); the nuclei of the medial and midline groups in general had a high density of calbindin neurons. Hypothalamic regions showed relatively high densities of calbindin neurons, particularly the ventromedial hypothalamic nucleus (6298 ± 736) and medial preoptic nucleus (8003 ± 975).

*Midbrain.* The superficial layers of the superior colliculus, grouped under the sensory-related superior colliculus in the CCFv3-2017 hierarchy was densely packed with calbindin neurons (3345 ± 430). This was in contrast to a relatively sparse distribution in the motor-related superior colliculus (480 ± 148). In general, midbrain areas were quite lightly stained for calbindin with low cell densities revealed in our analysis, although some areas stood out as densely packed with cells. This included the midbrain trigeminal nucleus (2209 ± 863), ventral tegmental area (4339 ± 914), paranigral area (2951 ± 620), periaqueductal gray (1270 ± 262), and midbrain raphe nuclei (1872 ± 371).

*Pons and medulla.* Pontine and medullary regions generally showed low to moderate densities of calbindin neurons. In the cerebellum, intense staining was seen in the Purkinje cells; however, because these cells were much larger than calbindin neurons in the rest of the brain, we did not obtain a satisfactory segmentation, and quantitative data are not presented.

### Different patterns of parvalbumin and calbindin neuron numbers in the mouse brain

**Figure 3:**
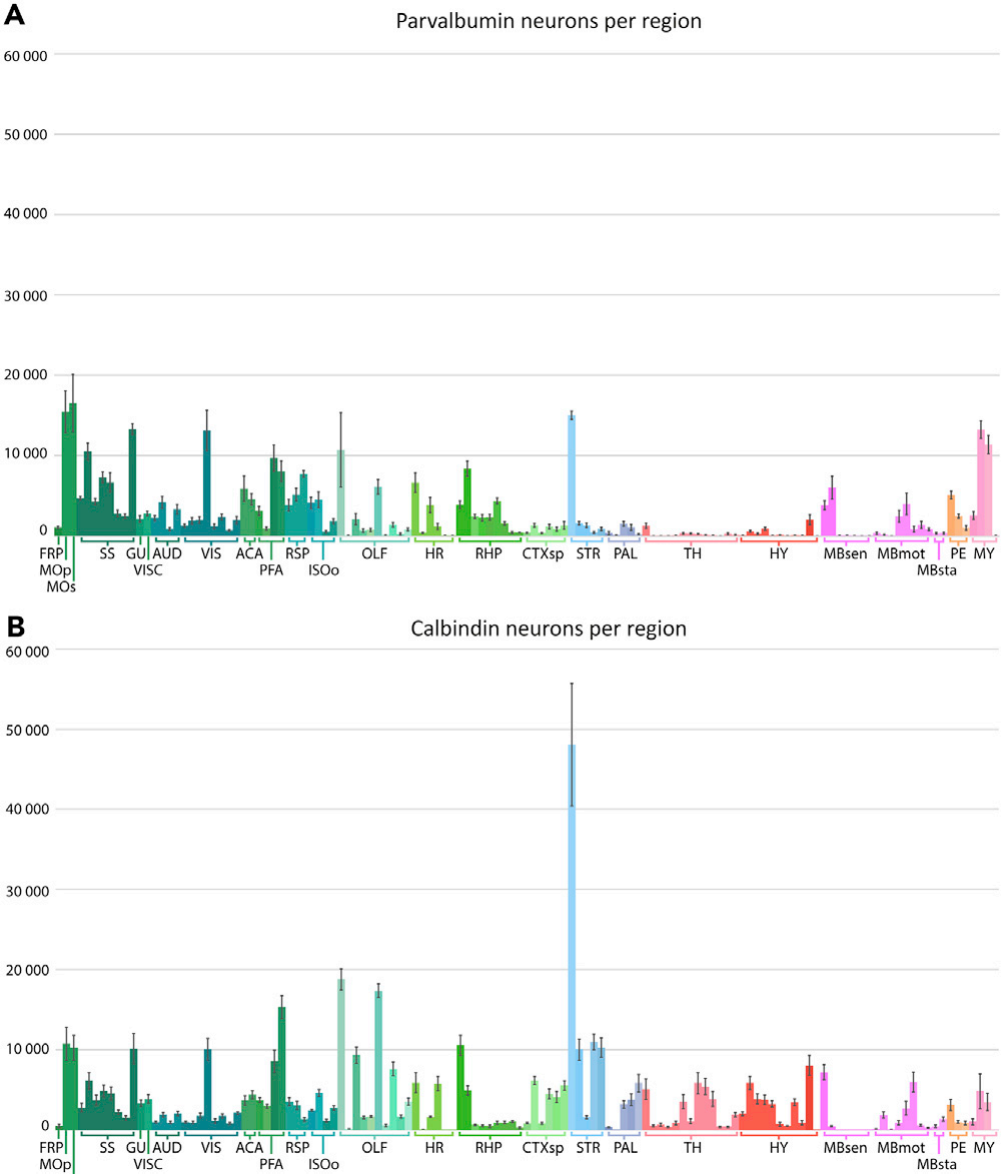
Comparison of total number estimates of parvalbumin and calbindin neurons across the mouse brain Bar chart showing mean bilateral total number estimates of parvalbumin (n = 4; (A)) and calbindin (n = 5, (B)) neurons in mouse brain regions. Regions are defined and color coded according to the CCFv3-2017. Error bars indicate SEM. Groups of brain areas are indicated along the x axis. Bars are placed from left to right in the same order as abbreviations are listed and explained in Figure 1. See Table S1 for an overview of all the total number estimates.

We compared total number estimates of parvalbumin and calbindin neurons across the mouse brain. Results per region for each cell type are shown in Figure 3. This comparison shows that the parvalbumin neurons generally outnumber calbindin neurons in isocortical and retrohippocampal areas. In contrast, the striatal, olfactory, and cortical subplate areas generally had higher numbers of calbindin neurons. Striking differences were seen in the thalamus and hypothalamus, where parvalbumin neurons were sparse (<500 parvalbumin neurons per mm^3^ across 10 out of 11 nuclei), whereas calbindin neurons showed high numbers in most subregions (>2000 calbindin cells per mm^3^ in 8 out of 11 nuclei). In midbrain areas, and most notably in the inferior colliculus and parabigeminal nucleus, the number of parvalbumin neurons exceeded that of calbindin neurons. In the pons and medulla, parvalbumin neurons showed the highest numbers as well; however, as mentioned, we grouped these regions quite broadly, and more fine-grained analysis would be needed to determine if smaller pontine and medullary nuclei might show different ratios of the two cell types.

**Figure 4:**
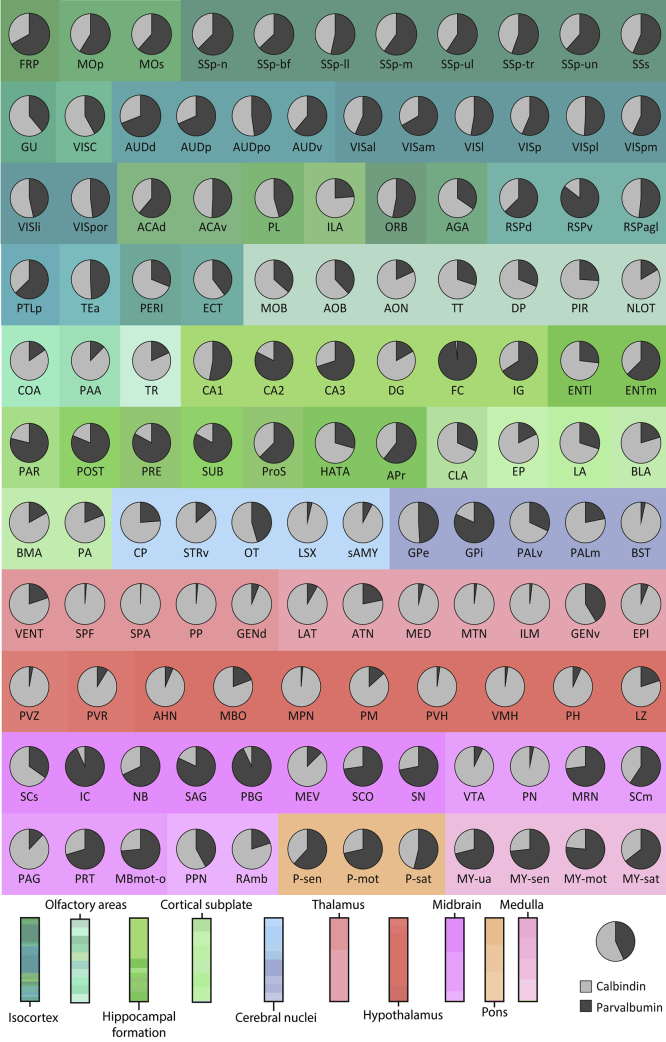
Ratios of parvalbumin and calbindin neurons across the mouse brain Pie charts showing the ratio of each cell type (calbindin in light gray, parvalbumin in dark gray) across mouse brain areas. Abbreviations are detailed in Figure 1.

To further explore the ratios of parvalbumin and calbindin neurons across the brain, we created pie charts showing the total number estimates for each cell type per region in the CCFv3-2017 (Figure 4). The figure clearly shows the trend that parvalbumin neurons were relatively more abundant in isocortical areas, particularly in somatosensory and motor cortical areas. However, in prefrontal cortices, e.g. prelimbic (PL), infralimbic (ILA), and agranular insular (AGA) areas, the balance was shifted toward more calbindin neurons. Calbindin neurons were also more numerous in gustatory (GU) and visceral (VISC) cortices, intercalated between somatosensory and olfactory cortical areas. Areas of the hippocampal and retrohippocampal regions also showed higher parvalbumin than calbindin neuron numbers, with the exceptions of the dentate gyrus (DG), lateral entorhinal area (ENTl), and hippocampo-amygdalar transition areas (HATA). In olfactory, striatal, and cortical subplate areas, the calbindin neurons were more abundant. The dorsal pallidal regions had relatively equal (GPe) or higher parvalbumin numbers (GPi), whereas the ventral and medial pallidum showed higher numbers of calbindin neurons. All nuclei of the thalamus and hypothalamus showed a higher number of calbindin than parvalbumin neurons. In the midbrain, pons, and medulla, parvalbumin neurons were generally more abundant than calbindin neurons. However, the pedunculopontine nucleus (PPN), sensory superior colliculus (SCs), paranigral area (PN), ventral tegmental area (VTA), midbrain trigeminal nucleus (MEV), periaqueductal gray (PAG), and raphe nuclei (RAmb) stood out with high calbindin neuron numbers relative to parvalbumin neurons.

### Comparative analysis of parvalbumin neurons in the rat and mouse hippocampal region

**Figure 5:**
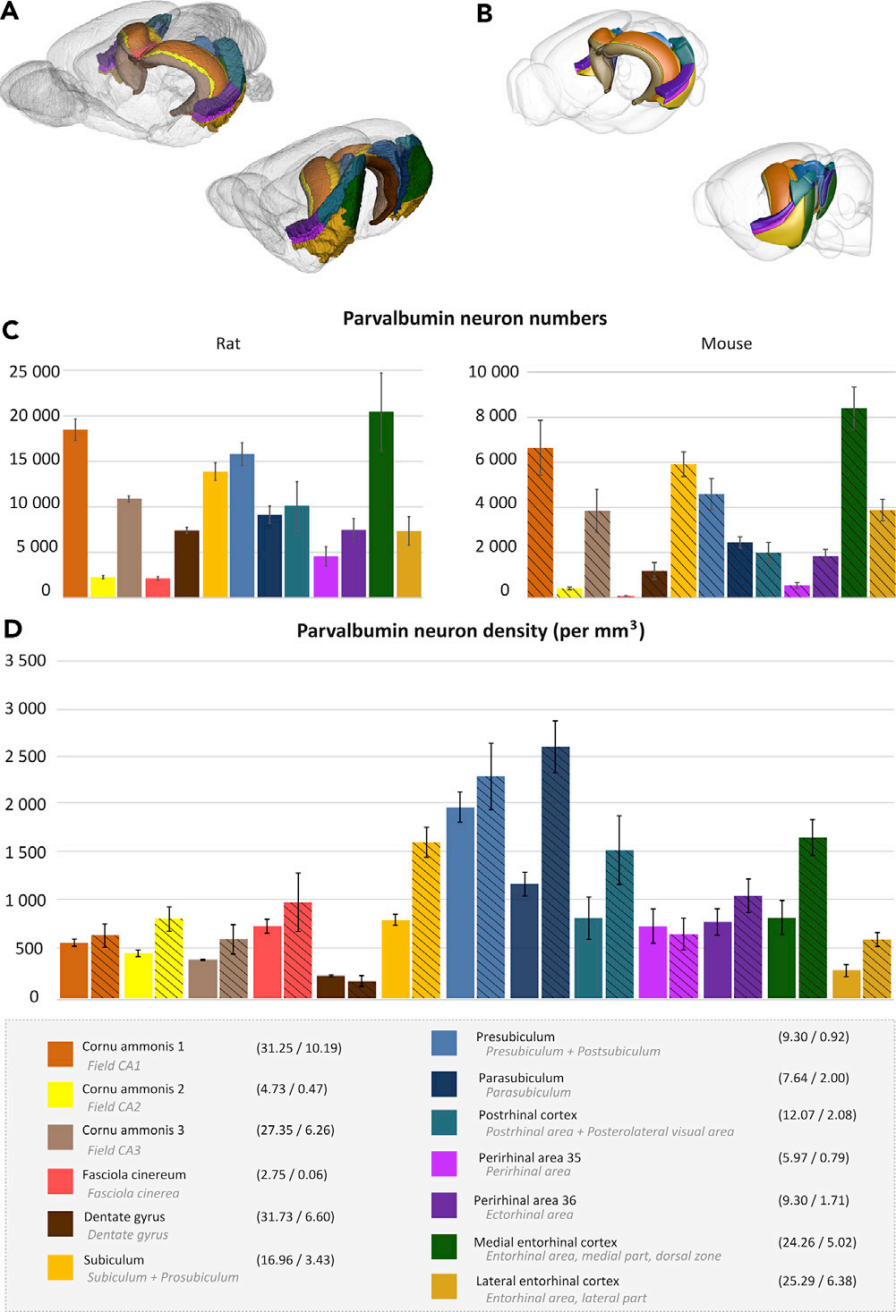
Cross-species comparison of parvalbumin neuron numbers and densities in hippocampal regions (A) Rostrolateral and caudolateral (with cerebellum and brainstem removed) 3D views of the hippocampal regions in the WHSv2, shown in the color they are assigned in the atlas (Kjonigsen et al., 2015) within a transparent view of the brain. (B) Corresponding 3D views of hippocampal regions in the CCFv3-2017. Regions are color coded according to their corresponding region in WHSv2. (C and D) (C) Bar graphs showing the mean bilateral total number of parvalbumin neurons in rat (left) and mouse (right) brain hippocampal regions. (D) Bar graph showing the density per mm^3^ of parvalbumin neurons in hippocampal regions. Solid bars show rat brain data and patterned bars show mouse brain data. Region names (WHSv2 terms in black text, corresponding CCFv3-2017 term in gray italic text), color codes according to WHSv2, and the volume (V) of each region (mouse/rat) are given in the lower panel. Error bars indicate SEM.

Parvalbumin neurons play an important role in the spatial circuits of the hippocampal region (Miao et al., 2017), where findings from rats and mice are often used interchangeably. To elucidate whether the parvalbumin neuron population in these species are similarly distributed within and across regions, we acquired immunohistochemical material showing parvalbumin neurons in the rat brain. We here compare the number, densities, and distributions of parvalbumin neurons in rat and mouse brain hippocampal regions. We focus on the hippocampal regions, as these are relatively similar among the two atlases used here (Kjonigsen et al., 2011; Wang et al., 2020). To facilitate comparison, we grouped regions in the CCFv3-2017 to their corresponding regions in the WHSv2 (Figures 5A and 5B). The brain regions mentioned in the following section are thus named according to the WHSv2 nomenclature and may differ slightly from CCFv3-2017 region terms used above. As collective terms, we use *hippocampal formation* to refer to regions of the Ammon's horn, dentate gyrus, fasciola cinereum and subiculum, and *parahippocampal region* for the pre- and parasubiculum and the entorhinal, perirhinal, and postrhinal cortices. These correspond to the collective terms hippocampal region and retrohippocampal region, respectively, in the CCFv3-2017. We use the term *hippocampal region* to refer to the hippocampal formation and parahippocampal region combined. Note that the medial entorhinal cortex is simply termed « entorhinal cortex» in WHSv2. We refer to it as medial entorhinal cortex in this manuscript, but it is called entorhinal cortex in the shared data files.

*Quantitative estimates of parvalbumin neurons in the rat and mouse brain hippocampal regions.* Our analysis of the hippocampal regions in the rat (n = 4) showed that the total number of parvalbumin neurons was highest in the medial entorhinal cortex (MEC; 20348 ± 4279), followed by cornu ammonis 1 (CA1; 18397 ± 1173), presubiculum (PrS; 15720 ± 1249), and subiculum (Sub; 13,804 ± 973). The highest density of parvalbumin neurons was seen in the PrS (1690 ± 134) and the parasubiculum (PaS; 1185 ± 125). The lateral entorhinal cortex (LEC) and dentate gyrus (DG) showed the lowest density of all subregions (LEC: 288 ± 62; DG: 231 ± 10). As in the rat, the mouse MEC had the highest number of parvalbumin neurons of all the hippocampal regions (8384 ± 940), followed by the CA1 (6631 ± 1221) and Sub (5898 ± 544). The density of parvalbumin neurons per mm^3^ was highest in the PaS (2614 ± 269) and PrS (2306 ± 346). The least dense parvalbumin neuron population was seen in the LEC (607 ± 75) and DG (178 ± 56). All total number and density estimates for the hippocampal regions of the rat and mouse are summarized in Figures 5C and 5D.

Thus, the estimated number of parvalbumin neurons was higher across all hippocampal regions of the rat brain as compared with the mouse—an expected finding given the relatively larger brain of the rat. In contrast, the density of parvalbumin neurons was generally higher in the mouse than in the rat (i.e. more parvalbumin neurons per mm^3^). Regions within the hippocampal formation were relatively similar in parvalbumin neuron density: compared with mice, rats showed 11%–35% lower density in regions of Ammon's horn (CA1-3), 30% higher density in the DG, and 25% lower in the fasciola cinereum (FC). Larger differences were seen in the parahippocampal regions, particularly in the PaS, the POR, and the entorhinal cortices, with rats having 46%–55% lower density as compared with the mice.

The relative density among regions in the hippocampal formation (including in CA1-3, DG, Sub, and the FC) was retained across species, with the highest density seen in Sub, followed by relatively similar densities in CA1-3, and noticeably lower density in the DG. The same was true for the parahippocampal regions, where the PrS and PaS regions showed the highest density of parvalbumin neurons of all hippocampal regions, followed by the MEC, perirhinal areas, and POR.

**Figure 6:**
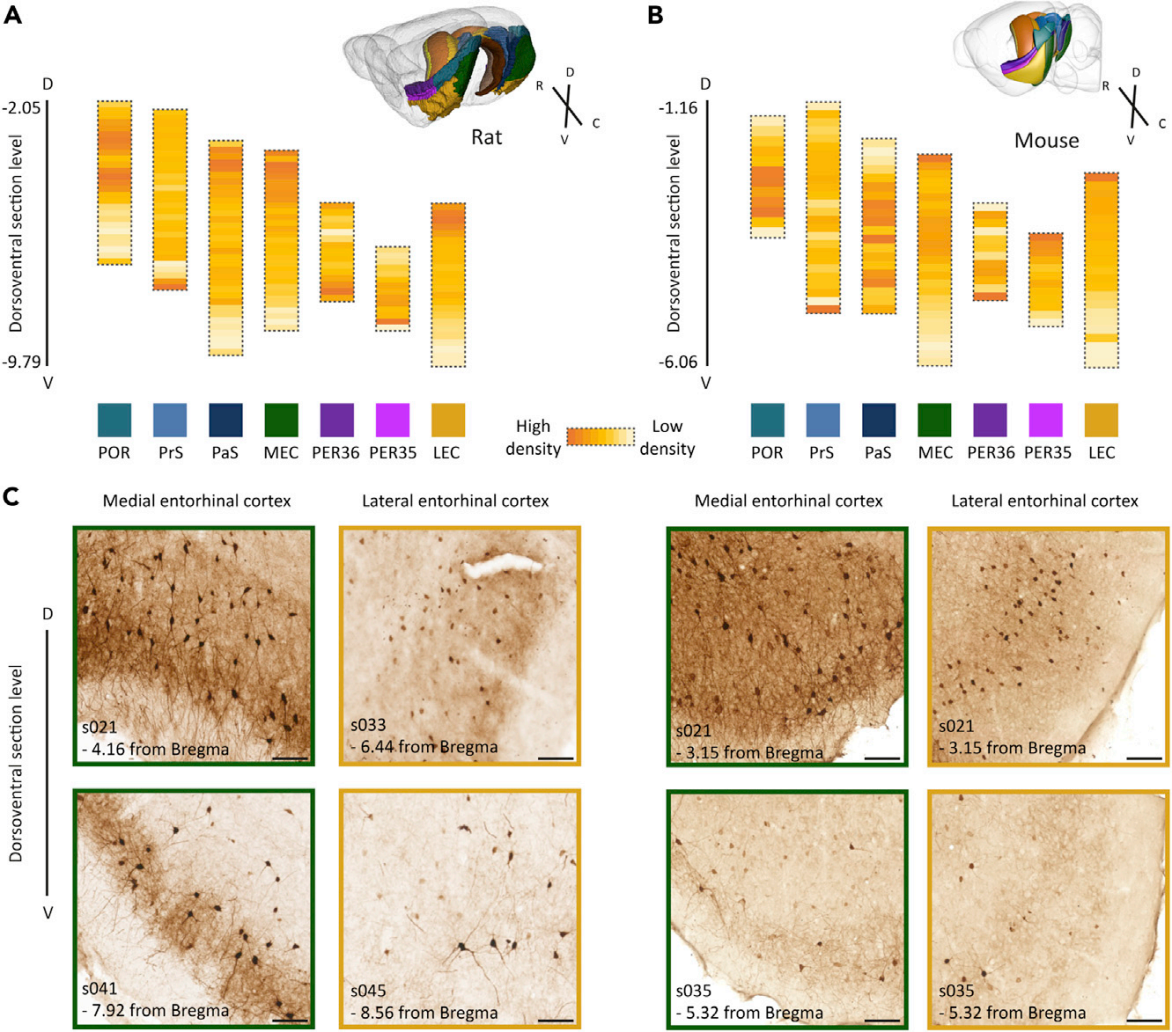
Parvalbumin neuron distribution along the dorsoventral axis of parahippocampal regions (A and B) Colored bars, each representing one area of the parahippocampal region, with individual segments in each bar corresponding to a section along the dorsoventral axis. Approximate Bregma positions of sections along the dorsoventral axis are given. Parvalbumin neuron density is indicated by the intensity of the color (from light yellow to dark orange).| (C) Example images from dorsal and ventral parts of the medial and lateral entorhinal cortex from rat (subject 25205, left panel) and mouse (subject 81266, right panel) showing a denser population of parvalbumin neurons in dorsal parts of these regions. Section numbers and approximate dorsoventral Bregma level are indicated for each image. Scale bars: 100 μm. Abbreviations: POR, postrhinal cortex; PrS, presubiculum; PaS, parasubiculum; MEC, medial entorhinal cortex; PER36, perirhinal area 36; PER35, perirhinal area 35; LEC, lateral entorhinal cortex.

*Distribution of parvalbumin within mouse and rat brain hippocampal regions.* Functional gradients are known to exist along the dorsoventral axis of the MEC (Brun et al., 2008; Giocomo et al., 2014) and in hippocampal-parahippocampal connectivity (Strange et al., 2014). We explored whether the density of parvalbumin neurons changed along the dorsoventral axis of each parahippocampal subregion. The results (summarized in Figure 6) indicated that the density of parvalbumin neurons decreases from dorsal to ventral levels in the POR, MEC, LEC, and PaS of the rat. We did not observe clear dorsoventral density gradients in the rat PrS or in the PER regions. In the mouse parahippocampal areas, the distribution of parvalbumin neurons decreased in the MEC and LEC, and a similar tendency was seen in perirhinal area 35 (PER35). No gradient was seen in the mouse PaS, but a dorsoventral increase in parvalbumin neuron density was observed in the mouse POR. However, this gradient was not seen when excluding the posterolateral visual area. In remaining areas, no clear gradients were observed (Figure 6).

### Comparison of findings to earlier published data

**Figure 7:**
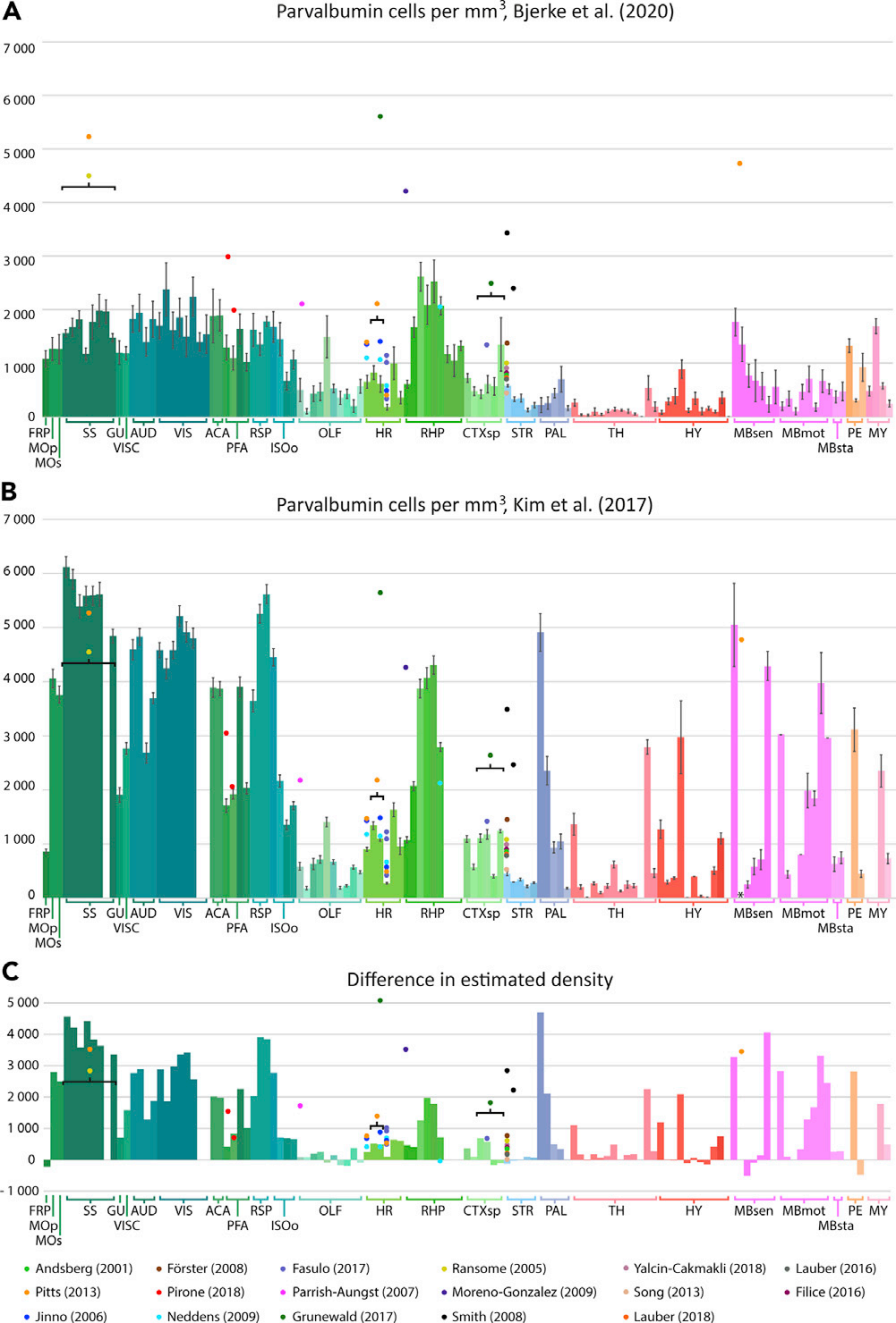
Comparison to previous reports (A) Mean parvalbumin neuron density per mm^3^ across the mouse brain reported here. (B) Mean parvalbumin neuron density per mm^3^ across the mouse brain as reported by Kim et al. (2017). Estimates (n = 32) found in 17 publications (listed below the bar graphs) are plotted in charts A and B according to the most closely matching region in the CCFv3-2017. Error bars indicate SEM. (C) Difference in density estimates reported by Kim et al. (2017) and in the current study (values from current study subtracted from values reported by Kim et al. (2017)). Literature values are plotted according to their difference from the current study (values from the current study subtracted from the values from literature studies). Error bars indicate SEM. Abbreviations are detailed in Figure 1. Literature references: Andsberg et al. (2001); Ransome and Turnley (2005); Jinno and Kosaka (2006); Parrish-Aungst et al. (2007); Smith et al. (2008); Förster (2008); Moreno-Gonzalez et al. (2009); Neddens and Buonanno (2009); Pitts et al. (2013); Song et al. (2013); Filice et al. (2016); Lauber et al. (2016), 2018; Fasulo et al. (2017); Grünewald et al. (2017); Pirone et al. (2018); Yalcin-Cakmakli et al. (2018). See Table S2 for detailed information on the literature sources.

We compared our estimates of parvalbumin neuron densities with the data presented by Kim et al. (2017) and with several other published reports (Figure 7). This comparison shows that our parvalbumin density estimates are generally lower than the ones provided by Kim et al. (2017). Differences between our estimates and those provided by Kim et al. (2017) were on average 57% in isocortical areas, 41% in olfactory areas, 38% in hippocampal regions, 35% in the cortical subplate, and 21% in the striatum. Larger differences were seen in the globus pallidus, small nuclei of the thalamus and hypothalamus, and in midbrain, pontine, and medullary nuclei where correspondence between atlas nomenclatures was poor. Still, many similar trends in the relative densities across regions can be seen in the datasets. We found 17 studies reporting parvalbumin neuron densities, in regions that could be mapped to closely corresponding region in the CCFv3-2017 atlas. Data for regions that could not be interpreted relative to the CCFv3-2017 atlas were not included in our comparison. Thirty-two estimates of parvalbumin densities were compared with our findings. The majority of these (19 of 32 estimates), mainly from hippocampal and striatal brain regions, were relatively well aligned with our density estimates, whereas the remaining ones (13 of 32 estimates) were considerably higher than the here reported estimates. All these studies used immunohistochemistry or fluorescence with antibodies targeting parvalbumin. A more detailed comparison (including information about strains, regions, and antibodies used in each study) can be found in Table S2, and similar comparison data for rat parvalbumin and mouse calbindin neurons can be found in Tables S3 and S4, respectively.

### Validity of QUINT results

Results obtained with the QUINT workflow critically depend on the validity of the segmentations used for quantification. To validate the numbers obtained using QUINT, i.e. counts of labeled neurons automatically detected using ilastik (below referred to as QUINT counts), we compared them with numbers obtained using manual identification of neurons (below referred to as manual counts).

#### Parvalbumin-stained material

We first compared numbers of parvalbumin positive neurons obtained in one hemisphere from one section and found that QUINT gave 9% higher counts in the neocortex (manual count = 2,175; QUINT count = 2379), 3% higher counts in the hippocampal and parahippocampal regions (manual count = 540, QUINT count = 555), and 15% higher counts in striatal and pallidal regions (manual count = 295, QUINT count = 340).

Secondly, we compared numbers obtained from the medial entorhinal cortex (23 sections) and found that QUINT provided a 5% higher total number of parvalbumin-positive neurons counted (manual count = 3,400; QUINT count = 3,560). Using Abercrombie's formula to correct for double-counting and multiplying by the section interval, the total number estimates in the unilateral medial entorhinal cortex were 14,836 using manual counts and 15,535 using QUINT counts.

For individual sections from the entorhinal cortex, we found the difference between QUINT counts and manual counts to be on average 5.5% (ranging from 0%–16% difference) between the two approaches. QUINT counts were generally higher than manual counts (in 21 of 23 sections), and the degree of difference was not associated with whether or not sections were used to train the ilastik segmentation algorithm (on average 5% difference in training sections and 6% difference in non-included sections). The results from both methods per section are summarized in Figure S1. By qualitative comparison of manually identified neurons and ilastik segmentations, we observed that the differences were mainly due to inclusion of labeled objects that were not cells (e.g. clustered neuropil) in the ilastik segmentation. In our main analysis, we therefore added a manual step to remove such erroneously segmented objects (see Transparent Methods for details).

#### Calbindin-stained material

We compared QUINT and manual counts of calbindin-positive neurons in one hemisphere in one section. QUINT counts were 10% higher in cortical regions (manual count = 654, QUINT count = 719) as well as in striatal and pallidal regions (manual count = 1,371, QUINT count = 1,513), whereas 2% lower in the hypothalamus (manual count = 98, QUINT count = 96). In olfactory regions, QUINT counts were 44% higher than the manual counts (manual count = 63, QUINT count = 91). We finally compared QUINT and manual counts of calbindin neurons in the anterior cingulate cortex and found that QUINT counts were 16% higher than manual counts (manual count = 905, QUINT count = 961). As for the parvalbumin data, we manually removed erroneously segmented objects from the calbindin image segmentations in our main analysis.

### Reliability of QUINT results

*Intrarater reliability.* To assess the intrarater reliability of the segmentations, one researcher trained three ilastik classifiers based on identical material to generate three sets of segmentations. Variability was very low in the hippocampus and parahippocampal areas (1%–3% difference) and neocortex (0.2%–2% difference); relatively low in the striatum (3%–10% difference), globus pallidus (1%–8% difference), and hypothalamus (10%–11% difference); and slightly higher in the substantia nigra (8–14% difference) and basal forebrain (3–13% difference). Results from the intrarater reliability test for all regions are summarized in Figure S2, and all the derived data are included in Data 1. The variability seemed independent of the time interval (a few days or six weeks) between the timing of the classifiers (Figure S2).

*Interrater reliability.* We assessed the interrater reliability of the segmentation results by presenting five researchers with the same material and the same instructions for segmentation. All the segmentations were then analyzed with the QUINT workflow based on identical atlas maps and the results compiled for major brain regions. Interrater reliability for these regions ranged from 8%–29% for the different researchers (see Data 1 and Figure S3 for a summary of the quantitative and qualitative results).

The segmentations generated by each researcher either consistently over- or underestimated the number of objects relative to the original segmentations that were used to produce the instructions, with the exception of two regions from one of the researchers. Qualitatively, we observed that the segmentations that underestimated objects relative to the original segmentation often did not extract the most “extreme” cases of labeling, i.e. very darkly stained cells or light-to-medium stained cells. The documentation instructed not to segment very light cells, which might have left room for interpretation in such cases.

To train pixel classifiers in ilastik, classes are created and example annotations applied to the image for each class. We exported and quantified the annotations placed by each researcher with the QUINT workflow using the default custom regions in Nutil Quantifier for the WHS atlas. The original classifier contained 90 annotated objects for the “cell” class, whereas the classifiers created as part of the interrater reliability study contained 33–63 annotations for this class. All the researchers placed “cell” annotations in the cortex and hippocampus, with all but one placing annotations in the olfactory and striatal/pallidal regions. For the “background” class, all the researchers placed annotations in the cortex, fiber tracts, and hippocampus, with most placing annotations in the olfactory regions as well. As the documentation instructed the researchers to place annotations in all these regions, there was some variability in the compliance to the segmentation instructions.

## Discussion

We have quantified densities and numbers of immunolabeled calbindin and parvalbumin neurons across the entire mouse brain using the QUINT workflow (Yates et al., 2019), which combines interactive machine-learning-based image segmentation with regions of interest defined using 3D anatomical references atlases. We also quantified parvalbumin neurons in the rat brain. We provide the first brain-wide quantitative analysis on the distribution of immunolabeled calbindin and parvalbumin cells. Below, we first discuss the efficiency, validity, and reproducibility of our methodology, with particular focus on our comparison to the literature, before elaborating on the quantitative results. Lastly, we discuss how open sharing of the different (raw and derived) components of our datasets may facilitate their re-use in new analyses.

Our methodology allows semi-automatic quantification of the numbers and densities of labeled neurons across the brain. The use of an open access 3D reference atlas makes it easier to compare data across studies, and open sharing of the different components of the datasets facilitates re-interpretation of results with different methods. The considerable variability in neuron numbers across the literature (Keller et al., 2018) and the challenges related to interpret the causes of such discrepancies (see, e.g. Bjerke et al., 2020a) highlights the need for re-usable data and transparent analyses. Additional studies will be needed to accumulate quantitative evidence about normal inter-individual variability.

We here used antibodies specific for parvalbumin or calbindin-expressing neurons, which have broad interest for their calcium-buffering capacities (Schwaller, 2020) and functional roles in various neural networks (Atallah et al., 2012; Li et al., 2017; Miao et al., 2017). Although immunohistochemistry is useful for mapping the distribution of cell types across entire brains, our protocol did not allow classification of cell subtypes. Subtypes are typically identified using combinations of morphological, electrophysiological, or hodological characteristics (see, e.g. Ascoli et al., 2008; Ibáñez-Sandoval et al., 2010). Our findings thus reflect broad classes of neurons, but does not distinguish cellular subtypes such as parvalbumin expressing basket or chandelier cells (Tremblay et al., 2016; Wang et al., 2019). Further studies are needed to map the distribution of finer neuronal subclasses. The QUINT workflow (Yates et al., 2019) is well suited for future studies replicating our findings and adding data for other markers, e.g. to address questions of variability within or across different strains, sex, or age groups. An important premise for such future comparisons is the use of the same anatomical reference atlas.

We have shown that the profile counts obtained with the QUINT workflow are in accordance with manual counts from the same areas. To further improve our ilastik segmentations, we implemented a post-processing step for the manual removal of erroneously segmented objects. To convert profile counts into cell number estimates for entire regions, it is necessary to address several sources of bias (Attili et al., 2019). First, when the brain is cut into sections, some cells are split and can therefore appear in more than one section, which may lead to overcounting. We corrected for this by using Abercrombie's formula (Abercrombie, 1946). Secondly, cells that are located at the border between two atlas regions must only be counted one. To ensure this, we switched off the “object splitting” feature in Nutil Quantifier to assign objects to one region only. Third, the bias of lost caps (i.e. cell fragments at the edges of a section may be "lost" or invisible; Hedreen, 1998) are inherent to profile counts in histological material. Correcting for this would require estimating a factor based on the observed number of profiles and the true number of cells, the latter that cannot be derived from section images. Lastly, cells located in deeper parts of a thick section may be occluded by those in the upper layer. Correcting for this would require dividing the section into layers along the z axis, which is also not possible in section images. Although we did not address these last two sources of bias, it has been shown that profile counting with Abercrombie's correction yields similar results to both stereology and three-dimensional reconstruction of entire cell populations (Baquet et al., 2009). Thus, to the extent to which our QUINT profile counts accurately reflect manual profile counts, we also consider them to reflect cell counts when corrected and multiplied to represent whole regions.

Our interrater reliability testing indicated that detailed instructions on the criteria for segmentation, with visualization of the expected outcome, can be effective in allowing researchers to recreate an analysis. In this test, only one parameter (the ilastik segmentations) was different between the researchers. In contrast, reproducibility of scientific findings is typically evaluated across scientific papers where the sources of variability may be many and hard to assess. Although methods sections in scientific reports are intended to provide sufficient and necessary details to reproduce results, they often lack critical information needed to interpret analyses (Bjerke et al., 2018; Keller et al., 2018). Our instructions were formulated as a stepwise procedure and may not be representative of a typical methods section, but we believe it can give clues to the details that are important for researchers to interpret and recreate an analysis. For documentation of counts, several visual examples of what is considered an object should be considered a minimum. Ideally, representations of objects across the entire material (such as the segmentation images provided here) allows other researchers to gain a deeper understanding of the analytic results.

We compared our brain-wide data on parvalbumin neuron densities with those provided by Kim et al. (2017) and found the densities obtained by Kim et al. (2017) to align well with our data in hippocampal and striatal regions, as well as in many thalamic and hypothalamic regions. Small differences can likely be ascribed to different definitions of the individual areas. Because Kim et al. (2017) employed a custom, 3D reconstructed version of the Allen Reference Atlas (Dong, 2008; Kim et al., 2015), we were not able to reliably compare data from all regions in their atlas version and ours. Significant changes were made to cortical and hippocampal delineations in the CCFv3-2017 delineations (Wang et al., 2020), and nomenclature differences in brainstem regions indicate that delineations in these regions have changed as well. For isocortical regions, however, Kim et al. (2017) reported density estimates that were much higher than ours across almost all regions. These differences are not likely to be caused only by different definitions of subregions. Although we used immunohistochemistry, Kim et al. (2017) employed Cre-reporter mice expressing fluorescent protein in parvalbumin neurons. As the two methods will visualize cells that express the parvalbumin protein and parvalbumin gene, respectively, our lower estimates could be caused by cells having only transient production of the protein, e.g. during development (Madisen et al., 2010) or variable expression levels associated with environmental/behavioral circumstances (Donato et al., 2013). Such cells would be detected by the Cre-reporter approach used by Kim et al. (2017) but not by immunohistochemistry as used in our study. Differences in segmentation and quantification methods may of course also influence results. Despite differences in data acquisition and analysis, similar trends in the relative densities of cells were seen across brain regions in the two datasets.

We also gathered quantitative estimates from the literature to benchmark our reported values. However, many studies report two- or three-dimensional densities based on one or a few sections that are unlikely to be representative for an entire region. Furthermore, regional areas or volumes estimated from sections will be highly affected by tissue shrinkage occurring during immunohistochemical procedures (Dorph-Petersen et al., 2001), and few studies report the use of shrinkage correction factors. In our analysis, we used the region volumes from the three-dimensional reference atlas, based on serial two-photon tomography and magnetic resonance imaging templates (for mouse and rat brain atlases, respectively). These are less affected by shrinkage than histological section material, which will result in estimated densities being lower. Given the large effects of tissue shrinkage on density estimates, it has been argued that total number estimates should preferentially be acquired and reported (Oorschot, 1994), but we observe that density estimates are more often reported in the literature. We note that the cell diameter measurement used in Abercrombie's formula in the current study will also be affected by tissue shrinkage, which will to some degree affect our total number estimates. Nevertheless, in the few cases where we found total number estimates from stereological studies with regions of interest closely corresponding to those used in our analysis (Andsberg et al., 2001; Filice et al., 2016; Lauber et al., 2016, 2018), we observed a high degree of correspondence with our total number estimates. This indicates that our estimates using Abercrombie's formula is not severely biased by the cell diameter approximation used here. Furthermore, for the literature sources that gave estimates from more than one region of interest, the same trend was seen across regions as in our data. We thus believe that the comparison of our parvalbumin data to those provided by Kim et al. (2017) taken together with those found in the other non-whole brain studies mentioned above indicates that our estimates reveal reproducible trends across regions.

For calbindin neurons in the mouse, very few quantitative estimates were available from the literature, some of which corresponded well to ours and some of which provided much higher numerical values. We note that a subset of calbindin neurons is very lightly stained (in accordance with previous observations, see Frantz and Tobin (1994)). The classifier used in our segmentation successfully extracted calbindin neurons of high and medium staining intensity but did not extract the most lightly stained neurons, thus our estimates might be considered lower bounds. Lightly stained neurons were seen across the brain but were most abundant in layer II of isocortical areas, in the striatum, the dentate gyrus, and hypothalamus. To extract these neurons, it might be necessary to train separate classifiers for different areas, perhaps also using images of higher resolution than used here. However, very light cells may be hard to distinguish from background staining also for a trained neuroanatomist.

Our comparison of calbindin and parvalbumin neuron numbers across the mouse brain revealed largely complementary patterns, possibly indicating differences in the relative contribution of these cell types within regions and across systems. The distribution of cells types, neurotransmitter receptors, and axonal connections varies substantially across different cortical and subcortical areas (see, e.g. Awasthi et al., 2020; Yu et al., 2019b). Such diversity occurs at multiple levels, from microcircuits to large-scale patterns across brain areas (Caroni, 2015; Kim et al., 2017), and specific combinations of multiple neurotransmitter receptors and cell types have been proposed to underlie specific functional characteristics of regions and networks (Zilles et al., 2015). In line with this, our findings show that parvalbumin neurons are more abundant in motor and sensory areas as well as in most of the hippocampal region, whereas calbindin neurons are dominant in limbic and hypothalamic areas. The importance of parvalbumin neurons in sensory-motor cortical areas was also highlighted by Kim et al. (2017), who found parvalbumin neurons to be the dominant among three interneuron types in these areas. They further found somatostatin-positive neurons to be the most prevalent interneuron type in cortical frontal and association areas. Calbindin may be expressed in subsets of somatostatin and vasointestinal protein expressing (VIP) neurons, which together with parvalbumin neurons make up almost all the interneurons in the neocortex (Rudy et al., 2011). However, calbindin is also known to be expressed in principal neurons, e.g. pyramidal cells in the CA1 (Merino-Serrais et al., 2020) and medial entorhinal cortex (Ohara et al., 2019; Ray et al., 2014). The thalamus also showed high numbers of calbindin neurons compared with parvalbumin positive ones. Given the recent finding that most thalamic nuclei have a very sparse GABAergic population (Evangelio et al., 2018), it is likely that the numerous calbindin neurons in the thalamus are excitatory principal neurons. In the midbrain, we found parvalbumin neurons to be dominant in sensory and motor-related regions such as the inferior colliculus, nucleus sagulum, parabigeminal nucleus, and substantia nigra, whereas calbindin was more prevalent in regions involved in behavioral state regulation and pain modulation, such as the periaqueductal gray, ventral tegmental area, and midbrain raphe nuclei. Thus, although calbindin-expressing neurons may represent both principal neurons and interneurons depending on the area in question, we observe that they generally seem to be most numerous in subcortical areas related to emotional processing and behavioral state regulation. Kim et al. found increased numbers of somatostatin and VIP neurons in several subcortical areas of female mice. Although our sample size of each sex in this study was insufficient to approach questions about sexual dimorphism, future studies may build on our material to allow such analyses. Together, our observations provide a neuroanatomical underpinning for recent evidence supporting the importance of calbindin neurons in social and anxiety-like behavior (Harris et al., 2016) and their susceptibility to stressful events (Li et al., 2017) and supports the already emphasized role of parvalbumin neurons in sensory systems and spatial navigation (Atallah et al., 2012; Miao et al., 2017; Runyan et al., 2010; Yu et al., 2016, 2019a).

In our cross-species comparison of parvalbumin neurons in the hippocampal region, we generally found higher parvalbumin neuron densities in mice than in rats, which is consistent with earlier reports of mice having lower total numbers but higher densities of neurons across the brain than rats (Herculano-Houzel et al., 2006). In both species, the density of parvalbumin neurons decreased from dorsal to ventral in the entorhinal cortex. This observation correlates with the increasing scaling of grid cell firing fields along the dorsoventral axis of the MEC (Brun et al., 2008; Stensola et al., 2012). Cells in the LEC have been found to be tuned to object positions and can coordinate to encode time information (Tsao et al., 2013, 2018), although the relationship between their properties and position along the dorsoventral axis is less well defined. A decreasing gradient in the inhibitory input from parvalbumin interneurons has been described from dorsal to ventral in the entorhinal cortex of mice (Beed et al., 2013; Kobro-Flatmoen and Witter, 2019), and dorsoventral gradients in the number of cell bodies have been described qualitatively in the LEC and MEC of adult mice (Fujimaru and Kosaka, 1996). Our evidence of a dorsal to ventral parvalbumin neuron density gradient in the MEC of both rats and mice correlates well with these connectional and functional gradients, and we show similar trends in the LEC as well. Recent research has indeed highlighted the possibility that similar principles govern the microcircuit wiring in MEC and LEC (Nilssen et al., 2018). Our results indicate that a decreasing dorsoventral density of parvalbumin interneurons may be one such principle.

Similarly, a decreasing density of parvalbumin neurons was seen from dorsal to ventral in the rat PaS and POR. The mouse POR did not show a dorsoventral decrease in parvalbumin neuron density; if anything, there was an opposite trend with increasing densities from dorsal to ventral. However, caution is warranted when interpreting this result, as no gradient was seen when excluding the region termed posterolateral visual area in the CCFv3-2017 from our definition of the mouse POR. A dorsoventral decrease in density was seen in the mouse PER35, although this gradient was not as clear as for the other areas mentioned. Several of the parahippocampal regions show a similar trend of decreasing parvalbumin neuron densities from dorsal to ventral, a trend that seemed more wide-spread in the rat. In conclusion, we show that parvalbumin neurons distribute according to similar principles in rat and mice hippocampal regions. Whether smaller differences between the species would persist across a larger sample and ultimately reflect functional specializations in rats and mice is a topic for future studies, but it should be noted that different configurations in a network might not necessarily critically affect function (Marder et al., 2015).

We share segmentation results together with the primary data from which they were derived. In addition to increasing transparency of analysis, this facilitates re-use and re-analysis of the derived data. For example, when new versions of the Waxholm Space rat brain atlas or the Allen Mouse Brain atlas are published, our segmentation images can be reanalyzed with new atlas maps. Thus, beyond the quantitative derived data presented here, we consider both the primary data and the segmentation maps to be re-usable for the community in the long term.

In conclusion, we here present numbers and distributions of parvalbumin and calbindin neurons across the mouse brain. We compare our results to previously published estimates, showing that our estimates of parvalbumin neurons across the mouse brain are well aligned with a previous brain-wide analysis (Kim et al., 2017) and the literature in striatal and hippocampal regions, where several studies have reported quantitative data. However, in other brain regions, larger differences were seen and very few studies were available. Direct comparisons are typically impeded by lack of information in publications, thus highlighting the need for transparent analyses and their reproduction. Furthermore, we compare the number and distribution of parvalbumin neurons in the mouse hippocampal region with similar data from the rat. Our analysis of parvalbumin and calbindin neurons points to trends within and across brain regions and species that align well with previous studies showing functional and connectional organization of these cell types.

### Limitations of the study

The limitations of our study are discussed at length in the main text. Notably, use of immunohistochemistry to identify parvalbumin and calbindin neurons may lead to different results than obtained with other methods for visualization of cells (e.g. the use of transgenic animals). Our method reveals broad cell classes and does not allow identification of subtypes such as parvalbumin positive basket and chandelier cells. Our use of atlas registration and semi-automated image analysis allowed efficient quantification across the brain, but the classification method did not necessarily capture all cells (e.g. lightly stained calbindin neurons). Results obtained should always be interpreted in light of the methodological approach, which might underlie some of the differences seen among studies in our comparison to the literature.

### Resource availability

#### Lead contact

For further information and requests for resources and reagents contact corresponding author, Trygve B. Leergaard (t.b.leergaard@medisin.uio.no).

#### Materials availability

This study did not generate new unique reagents.

#### Data and code availability

All raw and derived data from this project are shared via the EBRAINS research infrastructure (https://ebrains.eu/). The primary datasets contain high-resolution TIFF images of the immunohistochemical material and are shared under the following titles:

1) Distribution of calbindin-positive neurons in the normal adult mouse brain (Bjerke and Leergaard, 2020)
2) Distribution of parvalbumin-positive interneurons in the normal adult mouse brain (Laja et al., 2020a)
3) Distribution of parvalbumin-positive interneurons in the normal adult rat brain (Laja et al., 2020b)

The derived datasets contain downscaled PNG images of the primary data, PNG images for the segmentations, atlas maps (PNG and FLAT files), NUT files used to run Nutil Quantifier and all the output reports from this analysis, as well as the final quantitative results per region of interest as presented in this paper. The derived datasets are shared under the following titles:

1) Brain-wide quantitative data on calbindin-positive neurons in the mouse (Bjerke et al., 2020b)
2) Brain-wide quantitative data on parvalbumin-positive neurons in the mouse (Bjerke et al., 2020c)
3) Brain-wide quantitative data on parvalbumin-positive neurons in the rat (Bjerke et al., 2020d)

Together, the material provided in the derived dataset allows other researchers to re-run the analysis performed here, re-use the segmentation files with other atlas maps or other parameters in Nutil Quantifier, or re-segment the image material.

## Methods

All methods can be found in the accompanying Transparent Methods supplemental file.

## Supplemental information

Document S1. Transparent Methods, Figures S1–S4, and Tables S1–S4

Data S1. Data from Reliability Study, Related to Supplemental Figures 2 and 3

## References

[bib1] Abercrombie M. (1946). Estimation of nuclear population from microtome sections. Anat. Rec..

[bib2] Andsberg G., Kokaia Z., Lindvall O. (2001). Upregulation of p75 neurotrophin receptor after stroke in mice does not contribute to differential vulnerability of striatal neurons. Exp. Neurol..

[bib3] Arai R., Jacobowitz D., Deura S. (1994). Distribution of calretinin, calbindin-D28k, and parvalbumin in the rat thalamus. Brain Res. Bull..

[bib4] Ascoli G., Alonso-Nanclares L., Anderson S., Barrionuevo G., Benavides-Piccione R., Burkhalter A., Buzsáki G., Cauli B., Defelipe J., Fairén A. (2008). Petilla terminology: nomenclature of features of GABAergic interneurons of the cerebral cortex. Nat. Rev. Neurosci..

[bib5] Atallah B., Bruns W., Carandini M., Scanziani M. (2012). Parvalbumin-expressing interneurons linearly transform cortical responses to visual stimuli. Neuron.

[bib6] Attili S., Silva M., Nguyen T.-V., Ascoli G. (2019). Cell numbers, distribution, shape, and regional variation throughout the murine hippocampal formation from the adult brain Allen Reference Atlas. Brain Struct. Funct..

[bib7] Awasthi J.R., Tamada K., Overton E.T.N., Takumi T. (2020). Comprehensive topographical map of the serotonergic fibers in the male mouse brain. J. Comp. Neurol..

[bib8] Baquet Z., Williams D., Brody J., Smeyne R. (2009). A comparison of model-based (2D) and design-based (3D) stereological methods for estimating cell number in the substantia nigra pars compacta (SNpc) of the C57BL/6J mouse. Neuroscience.

[bib9] Beed P., Gundlfinger A., Schneiderbauer S., Song J., Böhm C., Burgalossi A., Brecht M., Vida I., Schmitz D. (2013). Inhibitory gradient along the dorsoventral axis in the medial entorhinal cortex. Neuron.

[bib10] Berg S., Kutra D., Kroeger T., Straehle C., Kausler B., Haubold C., Schiegg M., Ales J., Beier T., Rudy M. (2019). Ilastik: interactive machine learning for (bio)image analysis. Nat. Methods.

[bib11] Berridge M. (1998). Neuronal calcium signaling. Neuron.

[bib12] Bezaire M.J., Soltesz I. (2013). Quantitative assessment of CA1 local circuits: knowledge base for interneuron-pyramidal cell connectivity. Hippocampus.

[bib13] Bjerke I., Leergaard T. (2020). Distribution of calbindin positive neurons in the normal adult mouse brain. EBRAINS.

[bib14] Bjerke I., Øvsthus M., Andersson K., Blixhavn C., Kleven H., Yates S., Puchades M., Bjaalie J., Leergaard T. (2018). Navigating the murine brain: toward best practices for determining and documenting neuroanatomical locations in experimental studies. Front. Neuroanat..

[bib15] Bjerke I., Puchades M., Bjaalie J., Leergaard T. (2020). Database of literature derived cellular measurements from the murine basal ganglia. Sci. Data.

[bib16] Bjerke I., Yates S., Puchades M., Bjaalie J., Leergaard T. (2020). Brain-wide quantitative data on calbindin positive neurons in the mouse. EBRAINS.

[bib17] Bjerke I., Yates S., Puchades M., Bjaalie J., Leergaard T. (2020). Brain-wide quantitative data on parvalbumin positive neurons in the mouse. EBRAINS.

[bib18] Bjerke I., Yates S., Puchades M., Bjaalie J., Leergaard T. (2020). Brain-wide quantitative data on parvalbumin positive neurons in the rat. EBRAINS.

[bib19] Boccara C.N., Kjonigsen L.J., Hammer I.M., Bjaalie J.G., Leergaard T.B., Witter M.P. (2015). A three-plane architectonic atlas of the rat hippocampal region. Hippocampus.

[bib20] Brun V.H., Solstad T., Kjelstrup K.B., Fyhn M., Witter M.P., Moser E.I., Moser M.B. (2008). Progressive increase in grid scale from dorsal to ventral medial entorhinal cortex. Hippocampus.

[bib21] Caroni P. (2015). Inhibitory microcircuit modules in hippocampal learning. Curr. Opin. Neurobiol..

[bib22] Couey J.J., Witoelar A., Zhang S.J., Zheng K., Ye J., Dunn B., Czajkowski R., Moser M.B., Moser E.I., Roudi Y. (2013). Recurrent inhibitory circuitry as a mechanism for grid formation. Nat. Neurosci..

[bib23] Donato F., Rompani S.B., Caroni P. (2013). Parvalbumin-expressing basket-cell network plasticity induced by experience regulates adult learning. Nature.

[bib24] Dong H. (2008). Allen Reference Atlas: A Digital Color Brain Atlas of the C57BL/6J Male Mouse.

[bib25] Dorph-Petersen K.-A., Nyengaard J.R., Gundersen H.J.G. (2001). Tissue shrinkage and unbiased stereological estimation of particle number and size. J. Microsc..

[bib26] Evangelio M., García-Amado M., Clascá F. (2018). Thalamocortical projection neuron and interneuron numbers in the visual thalamic nuclei of the adult C57BL/6 mouse. Front. Neuroanat..

[bib27] Fasulo L., Brandi R., Arisi I., La Regina F., Berretta N., Capsoni S., D’Onofrio M., Cattaneo A. (2017). ProNGF drives localized and cell selective parvalbumin interneuron and perineuronal net depletion in the dentate gyrus of transgenic mice. Front. Mol. Neurosci..

[bib28] Ferguson B., Gao W. (2018). PV interneurons: critical regulators of E/I balance for prefrontal cortex-dependent behavior and psychiatric disorders. Front. Neural Circuits.

[bib29] Filice F., Vörckel K.J., Sungur A.Ö., Wöhr M., Schwaller B. (2016). Reduction in parvalbumin expression not loss of the parvalbumin-expressing GABA interneuron subpopulation in genetic parvalbumin and shank mouse models of autism. Mol. Brain.

[bib30] Förster J. (2008). Quantitative Morphological Analysis of the Neostriatum and the Cerebellum of Tenascin-C Deficient Mice. http://citeseerx.ist.psu.edu/viewdoc/download?doi=10.1.1.427.6474&rep=rep1&type=pdf.

[bib31] Frantz G., Tobin A. (1994). Cellular distribution of calbindin D28K mRNAs in the adult mouse brain. J. Neurosci. Res..

[bib32] Fujimaru Y., Kosaka T. (1996). The distribution of two calcium binding proteins, calbindin D-28K and parvalbumin, in the entorhinal cortex of the adult mouse. Neurosci. Res..

[bib33] Giocomo L., Stensola T., Bonnevie T., Van Cauter T., Moser M., Moser E. (2014). Topography of head direction cells in medial entorhinal cortex. Curr. Biol..

[bib34] Gogolla N., LeBlanc J., Quast K., Südhof T., Fagiolini M., Hensch T. (2009). Common circuit defect of excitatory-inhibitory balance in mouse models of autism. J. Neurodev. Disord..

[bib35] Gonzalez-Burgos G., Lewis D. (2012). NMDA receptor hypofunction, parvalbumin-positive neurons, and cortical gamma oscillations in schizophrenia. Schizophr. Bull..

[bib36] Groeneboom N., Yates S., Puchades M., Bjaalie J. (2020). Nutil: a pre- and post-processing toolbox for histological rodent brain section images. Front. Neuroinform..

[bib37] Grünewald B., Lange M.D., Werner C., O’Leary A., Weishaupt A., Popp S., Pearce D.A., Wiendl H., Reif A., Pape H.C. (2017). Defective synaptic transmission causes disease signs in a mouse model of juvenile neuronal ceroid lipofuscinosis. Elife.

[bib38] Hafting T., Fyhn M., Molden S., Moser M.B., Moser E.I. (2005). Microstructure of a spatial map in the entorhinal cortex. Nature.

[bib39] Harris E., Abel J., Tejada L., Rissman E. (2016). Calbindin knockout alters sex-specific regulation of behavior and gene expression in amygdala and prefrontal cortex. Endocrinology.

[bib40] Hashimoto T., Volk D., Eggan S., Mirnics K., Pierri J., Sun Z., Sampson A., Lewis D. (2003). Gene expression deficits in a subclass of GABA neurons in the prefrontal cortex of subjects with schizophrenia. J. Neurosci..

[bib41] Hedreen J.C. (1998). Lost caps in histological counting methods. Anat. Rec..

[bib42] Herculano-Houzel S., Mota B., Lent R. (2006). Cellular scaling rules for rodent brains. Proc. Natl. Acad. Sci. U S A.

[bib43] Hu H., Gan J., Jonas P. (2014). Fast-spiking, parvalbumin^+^ GABAergic interneurons: from cellular design to microcircuit function. Science.

[bib44] Ibáñez-Sandoval O., Tecuapetla F., Unal B., Shah F., Koós T., Tepper J.M. (2010). Electrophysiological and morphological characteristics and synaptic connectivity of tyrosine hydroxylase-expressing neurons in adult mouse striatum. J. Neurosci..

[bib45] Jinno S., Kosaka T. (2006). Cellular architecture of the mouse hippocampus: a quantitative aspect of chemically defined GABAergic neurons with stereology. Neurosci. Res..

[bib46] Kalanithi P., Zheng W., Kataoka Y., DiFiglia M., Grantz H., Saper C., Schwartz M., Leckman J., Vaccarino F. (2005). Altered parvalbumin-positive neuron distribution in basal ganglia of individuals with Tourette syndrome. Proc. Natl. Acad. Sci. U S A.

[bib47] Keller D., Erö C., Markram H. (2018). Cell densities in the mouse brain: a systematic review. Front. Neuroanat..

[bib48] Kim Y., Venkataraju K.U., Pradhan K., Mende C., Taranda J., Turaga S.C., Arganda-Carreras I., Ng L., Hawrylycz M.J., Rockland K.S. (2015). Mapping social behavior-induced brain activation at cellular resolution in the mouse. Cell Rep..

[bib49] Kim Y., Yang G., Pradhan K., Venkataraju K., Bota M., García del Molino L., Fitzgerald G., Ram K., He M., Levine J. (2017). Brain-wide maps reveal stereotyped cell-type-based cortical architecture and subcortical sexual dimorphism. Cell.

[bib50] Kjonigsen L., Lillehaug S., Bjaalie J., Witter M., Leergaard T. (2015). Waxholm Space atlas of the rat brain hippocampal region: three-dimensional delineations based on magnetic resonance and diffusion tensor imaging. Neuroimage.

[bib51] Kjonigsen L.J., Leergaard T.B., Witter M.P., Bjaalie J.G. (2011). Digital atlas of anatomical subdivisions and boundaries of the rat hippocampal region. Front. Neuroinform..

[bib52] Kobro-Flatmoen A., Witter M. (2019). Neuronal chemo-architecture of the entorhinal cortex: a comparative review. Eur. J. Neurosci..

[bib53] Laja A., Bjerke I., Leergaard T., Witter M. (2020). Distribution of parvalbumin-positive interneurons in the normal adult mouse brain. EBRAINS.

[bib54] Laja A., Bjerke I., Leergaard T., Witter M. (2020). Distribution of parvalbumin-positive interneurons in the normal adult rat brain. EBRAINS.

[bib55] Lauber E., Filice F., Schwaller B. (2016). Prenatal valproate exposure differentially affects parvalbumin-expressing neurons and related circuits in the cortex and striatum of mice. Front. Mol. Neurosci..

[bib56] Lauber E., Filice F., Schwaller B. (2018). Dysregulation of parvalbumin expression in the Cntnap2 −/− mouse model of autism spectrum disorder. Front. Mol. Neurosci..

[bib57] Li J., Xie X., Yu J., Sun Y., Liao X., Wang X., Su Y., Liu Y., Schmidt M., Wang X. (2017). Suppressed calbindin levels in hippocampal excitatory neurons mediate stress-induced memory loss. Cell Rep..

[bib58] Lu E., Llano D.A., Sherman S.M. (2009). Different distributions of calbindin and calretinin immunostaining across the medial and dorsal divisions of the mouse medial geniculate body. Hear. Res..

[bib59] Madisen L., Zwingman T.A., Sunkin S.M., Oh S.W., Zariwala H.A., Gu H., Ng L.L., Palmiter R.D., Hawrylycz M.J., Jones A.R. (2010). A robust and high-throughput Cre reporting and characterization system for the whole mouse brain. Nat. Neurosci..

[bib60] Marder E., Goeritz M.L., Otopalik A.G. (2015). Robust circuit rhythms in small circuits arise from variable circuit components and mechanisms. Curr. Opin. Neurobiol..

[bib61] Marín O. (2012). Interneuron dysfunction in psychiatric disorders. Nat. Rev. Neurosci..

[bib62] Markram H., Toledo-Rodriguez M., Wang Y., Gupta A., Silberberg G., Wu C. (2004). Interneurons of the neocortical inhibitory system. Nat. Rev. Neurosci..

[bib63] Merino-Serrais P., Tapia-González S., DeFelipe J. (2020). Calbindin immunostaining in the CA1 hippocampal pyramidal cell layer of the human and mouse: a comparative study. J. Chem. Neuroanat..

[bib64] Miao C., Cao Q., Moser M., Moser E. (2017). Parvalbumin and somatostatin interneurons control different space-coding networks in the medial entorhinal cortex. Cell.

[bib65] Moreno-Gonzalez I., Baglietto-Vargas D., Sanchez-Varo R., Jimenez S., Trujillo-Estrada L., Sanchez-Mejias E., Del Rio J.C., Torres M., Romero-Acebal M., Ruano D. (2009). Extracellular amyloid-β and cytotoxic glial activation induce significant entorhinal neuron loss in young PS1M146L/APP751SL mice. J. Alzheimer’s Dis..

[bib66] Murakami T., Mano T., Saikawa S., Horiguchi S., Shigeta D., Baba K., Sekiya H., Shimizu Y., Tanaka K., Kiyonari H. (2018). A three-dimensional single-cell-resolution whole-brain atlas using CUBIC-X expansion microscopy and tissue clearing. Nat. Neurosci..

[bib67] Neddens J., Buonanno A. (2009). Selective populations of hippocampal interneurons express ErbB4 and their number and distribution is altered in ErbB4 knockout mice. Hippocampus.

[bib68] Nilssen E., Jacobsen B., Fjeld G., Nair R., Blankvoort S., Kentros C., Witter M. (2018). Inhibitory connectivity dominates the fan cell network in layer II of lateral entorhinal cortex. J. Neurosci..

[bib69] Ohara S., Gianatti M., Itou K., Berndtsson C.H., Doan T.P., Kitanishi T., Mizuseki K., Iijima T., Tsutsui K.-I., Witter M.P. (2019). Entorhinal layer II calbindin-expressing neurons originate widespread telencephalic and intrinsic projections. Front. Syst. Neurosci..

[bib70] Oorschot D. (1994). Are you using neuronal densities, synaptic densities or neurochemical densities as your definitive data? There is a better way to go. Prog. Neurobiol..

[bib71] Papp E., Leergaard T., Calabrese E., Johnson G., Bjaalie J. (2014). Waxholm Space atlas of the Sprague dawley rat brain. Neuroimage.

[bib72] Parrish-Aungst S., Shipley M.T., Erdelyi F., Szabo G., Puche A.C. (2007). Quantitative analysis of neuronal diversity in the mouse olfactory bulb. J. Comp. Neurol..

[bib73] Pirone A., Alexander J., Koenig J., Cook-Snyder D., Palnati M., Wickham R., Eden L., Shrestha N., Reijmers L., Biederer T. (2018). Social stimulus causes aberrant activation of the medial prefrontal cortex in a mouse model with autism-like behaviors. Front. Synaptic Neurosci..

[bib74] Pitts M.W., Reeves M.A., Hashimoto A.C., Ogawa A., Kremer P., Seale L.A., Berry M.J. (2013). Deletion of selenoprotein M leads to obesity without cognitive deficits. J. Biol. Chem..

[bib75] Puchades M., Csucs G., Ledergerber D., Leergaard T., Bjaalie J. (2019). Spatial registration of serial microscopic brain images to three-dimensional reference atlases with the QuickNII tool. PLoS One.

[bib76] Ransome M.I., Turnley A.M. (2005). Analysis of neuronal subpopulations in mice over-expressing suppressor of cytokine signaling-2. Neuroscience.

[bib77] Ray S., Naumann R., Burgalossi A., Tang Q., Schmidt H., Brecht M. (2014). Grid-layout and theta-modulation of layer 2 pyramidal neurons in medial entorhinal cortex. Science.

[bib78] Rogers J.H., Résibois A. (1992). Calretinin and calbindin-D28k in rat brain: patterns of partial co-localization. Neuroscience.

[bib79] Rudy B., Fishell G., Lee S., Hjerling-Leffler J. (2011). Three groups of interneurons account for nearly 100% of neocortical GABAergic neurons. Dev. Neurobiol..

[bib80] Runyan C.A., Schummers J., Van Wart A., Kuhlman S.J., Wilson N.R., Huang Z.J., Sur M. (2010). Response features of parvalbumin-expressing interneurons suggest precise roles for subtypes of inhibition in visual cortex. Neuron.

[bib81] Schmid J.S., Bernreuther C., Nikonenko A.G., Ling Z., Mies G., Hossmann K.A., Jakovcevski I., Schachner M. (2013). Heterozygosity for the mutated X-chromosome-linked L1 cell adhesion molecule gene leads to increased numbers of neurons and enhanced metabolism in the forebrain of female carrier mice. Brain Struct. Funct..

[bib82] Schwaller B. (2010). Cytosolic Ca2+ buffers. Cold Spring Harb. Perspect. Biol..

[bib83] Schwaller B. (2020). Cytosolic Ca2+ buffers are inherently Ca2+ signal modulators. Cold Spring Harb. Perspect. Biol..

[bib84] Silvestri L., Paciscopi M., Soda P., Biamonte F., Iannello G., Frasconi P., Pavone F. (2015). Quantitative neuroanatomy of all Purkinje cells with light sheet microscopy and high-throughput image analysis. Front. Neuroanat..

[bib85] Smith K.M., Fagel D.M., Stevens H.E., Maragnoli M.E., Rabenstein R.L., Ohkubo Y., Picciotto M.R., Schwartz M.L., Vaccarino F.M. (2008). Deficiency in inhibitory cortical interneurons associates with hyperactivity in fibroblast growth factor receptor 1 mutant mice. Biol. Psychiatry.

[bib86] Song C.-H., Bernhard D., Bolarinwa C., Hess E.J., Smith Y., Jinnah H.A. (2013). Subtle microstructural changes of the striatum in a DYT1 knock-in mouse model of dystonia. Neurobiol. Dis..

[bib87] Stensola H., Stensola T., Solstad T., Frøland K., Moser M., Moser E. (2012). The entorhinal grid map is discretized. Nature.

[bib88] Strange B.A., Witter M.P., Lein E.S., Moser E.I. (2014). Functional organization of the hippocampal longitudinal axis. Nat. Rev. Neurosci..

[bib89] Sun S., Li F., Gao X., Zhu Y., Chen J., Zhu X., Yuan H., Gao D. (2011). Calbindin-D28K inhibits apoptosis in dopaminergic neurons by activation of the PI3-kinase-Akt signaling pathway. Neuroscience.

[bib90] Szabadics J., Varga C., Brunner J., Chen K., Soltesz I. (2010). Granule cells in the CA3 area. J. Neurosci..

[bib91] Tremblay R., Lee S., Rudy B. (2016). GABAergic interneurons in the neocortex: from cellular properties to circuits. Neuron.

[bib92] Tsao A., Moser M.-B., Moser E.I. (2013). Traces of experience in the lateral entorhinal cortex. Curr. Biol..

[bib93] Tsao A., Sugar J., Lu L., Wang C., Knierim J., Moser M., Moser E. (2018). Integrating time from experience in the lateral entorhinal cortex. Nature.

[bib94] Wang Q., Ding S., Li Y., Royall J., Feng D., Lesnar P., Graddis N., Naeemi M., Facer B., Ho A. (2020). The allen mouse brain Common coordinate Framework: a 3D reference atlas. Cell.

[bib95] Wang X., Tucciarone J., Jiang S., Yin F., Wang B.S., Wang D., Jia Y., Jia X., Li Y., Yang T. (2019). Genetic single neuron anatomy reveals fine granularity of cortical axo-axonic cells. Cell Rep..

[bib96] Yalcin-Cakmakli G., Rose S.J., Villalba R.M., Williams L., Jinnah H.A., Hess E.J., Smith Y. (2018). Striatal cholinergic interneurons in a knock-in mouse model of L -DOPA-Responsive dystonia. Front. Syst. Neurosci..

[bib97] Yates S., Groeneboom N., Coello C., Lichtenthaler S., Kuhn P.-H., Demuth H.-U., Hartlage-Rübsamen M., Roßner S., Leergaard T., Kreshuk A. (2019). QUINT: workflow for quantification and spatial analysis of features in histological images from rodent brain. Front. Neuroinform..

[bib98] Yu J., Gutnisky D.A., Hires S.A., Svoboda K. (2016). Layer 4 fast-spiking interneurons filter thalamocortical signals during active somatosensation. Nat. Neurosci..

[bib99] Yu J., Hu H., Agmon A., Svoboda K. (2019). Recruitment of GABAergic interneurons in the barrel cortex during active tactile behavior. Neuron.

[bib100] Yu Q., Liu Y.Z., Zhu Y.B., Wang Y.Y., Li Q., Yin D.M. (2019). Genetic labeling reveals temporal and spatial expression pattern of D2 dopamine receptor in rat forebrain. Brain Struct. Funct..

[bib101] Zhang C., Yan C., Ren M., Li A., Quan T., Gong H. (2017). A platform for stereological quantitative analysis of the brain- wide distribution of type-specific neurons. Sci. Rep..

[bib102] Zilles K., Bacha-Trams M., Palomero-Gallagher N., Amunts K., Friederici A.D. (2015). Common molecular basis of the sentence comprehension network revealed by neurotransmitter receptor fingerprints. Cortex.

