# Supplementary material for: Densities and numbers of calbindin and parvalbumin positive neurons across the rat and mouse brain
Source: iScience. 2020 Dec 8;24(1):101906. doi: 10.1016/j.isci.2020.101906 (PMC7770605; doi:10.1016/j.isci.2020.101906)
Supplement: Document S1. Transparent Methods, Figures S1–S4, and Tables S1–S4 [file mmc1.pdf]

iScience, Volume *24*

Supplemental Information

Densities and numbers of calbindin and parvalbumin positive neurons across the rat and mouse brain

Ingvild E. Bjerke, Sharon C. Yates, Arthur Laja, Menno P. Witter, Maja A. Puchades, Jan G. Bjaalie, and Trygve B. Leergaard

Supplemental Figure 1. Comparison of manual and QUINT counts of parvalbumin positive profiles across the medial entorhinal cortex. Related to Transparent Methods, "Validity of segmentations".

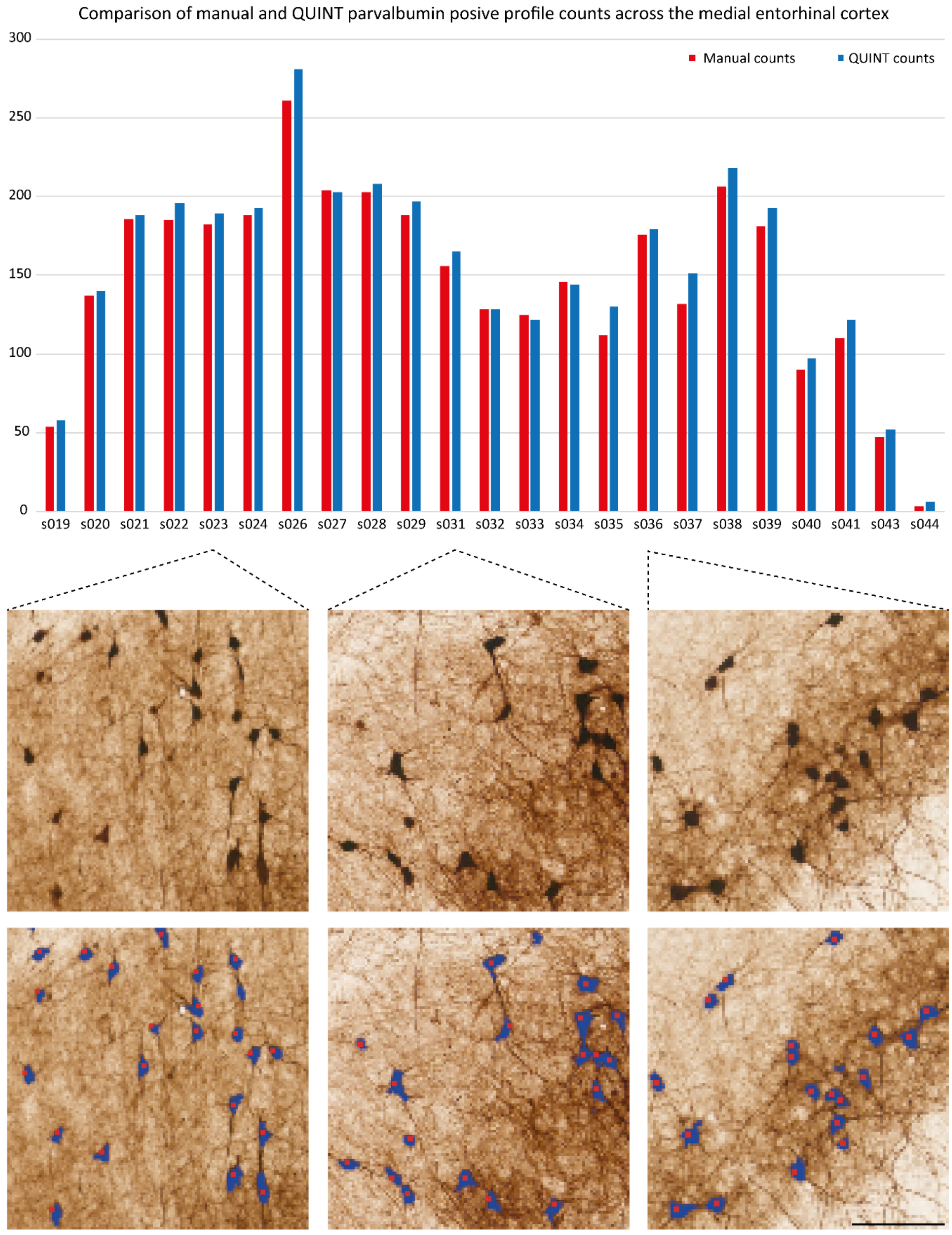

The graph shows manual counts (red bars) and QUINT counts (blue bars) from horizontal sections throughout the medial entorhinal cortex. Bottom row shows example of manual and ilastik segmentations overlayed to the parvalbumin stained material. Scale bar: 100 µm.

Supplemental Figure 2. Reliability of segmentations from one researcher. Related to Transparent Methods, "Reliability of segmentations".

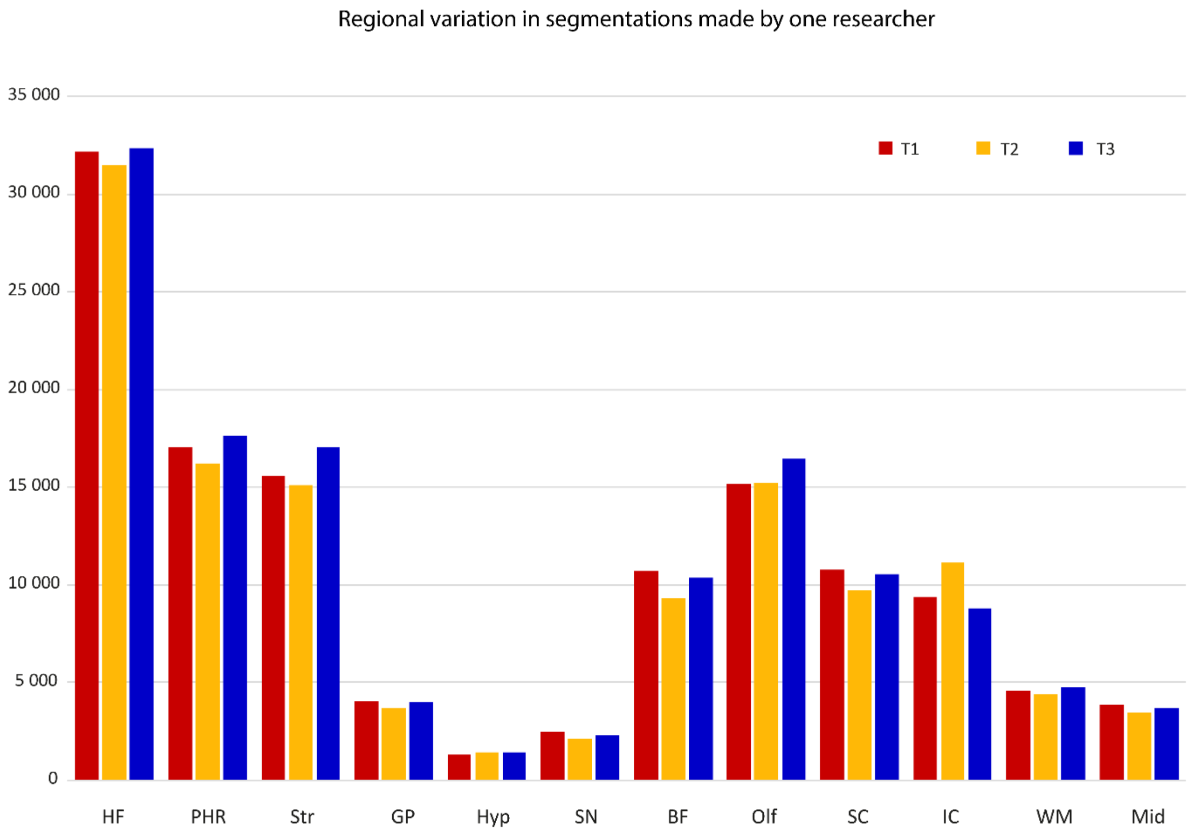

Bar chart showing the results from quantification of segmentations made by one researcher at three different time points (T1-3). Abbreviations: BF, basal forebrain; GP, globus pallidus; HF, hippocampal formation; Hyp, hypothalamus; IC, inferior colliculus; PHR, parahippocampal region; Mid, other midbrain regions Str, striatum; SN, substantia nigra; Olf, olfactory regions; SC, superior colliculus; WM, white matter. A list of which regions of the Waxholm Space rat brain atlas that are grouped in these abbreviations is given in Supplemental Data 1.

Supplemental Figure 3. Reliability of segmentations across researchers. Related to Transparent Methods, “Reliability of segmentations”.

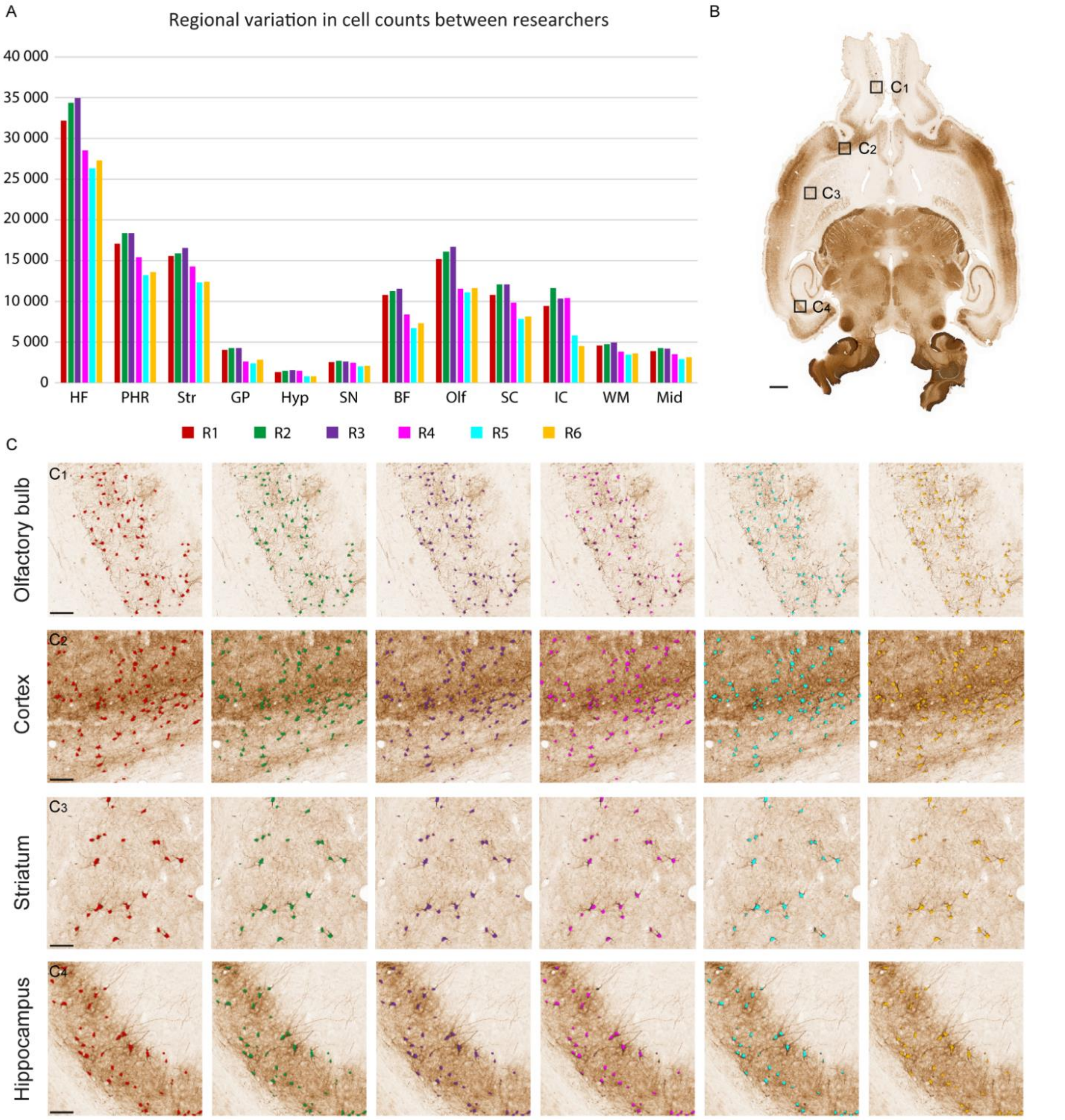

(A) Bar chart showing variations between different researchers segmentation results. One researcher (R1, red bars) performed a segmentation and provided documentation (see, Transparent methods) for replication of the analysis by researchers 2-5. Examples from the segmentations produced by each researcher are shown as overlays on the original images in the lower panel (C1-C4, colour coded in accordance with the bar graph). Scale bars are 1 mm in (B) and 100 µm in C1-C4. Abbreviations: BF, basal forebrain; GP, globus pallidus; HF, hippocampal formation; Hyp, hypothalamus; IC, inferior colliculus; PHR, parahippocampal region; Mid, other midbrain regions Str, striatum; SN, substantia nigra; Olf, olfactory regions; SC, superior colliculus; WM, white matter. A list of which regions of the Waxholm Space rat brain atlas that are grouped in these abbreviations is given in Supplemental Data 1.

Supplemental Figure 4. Examples of images with atlas overlays and segmentation results. Related to Transparent Methods, “Image segmentation”.

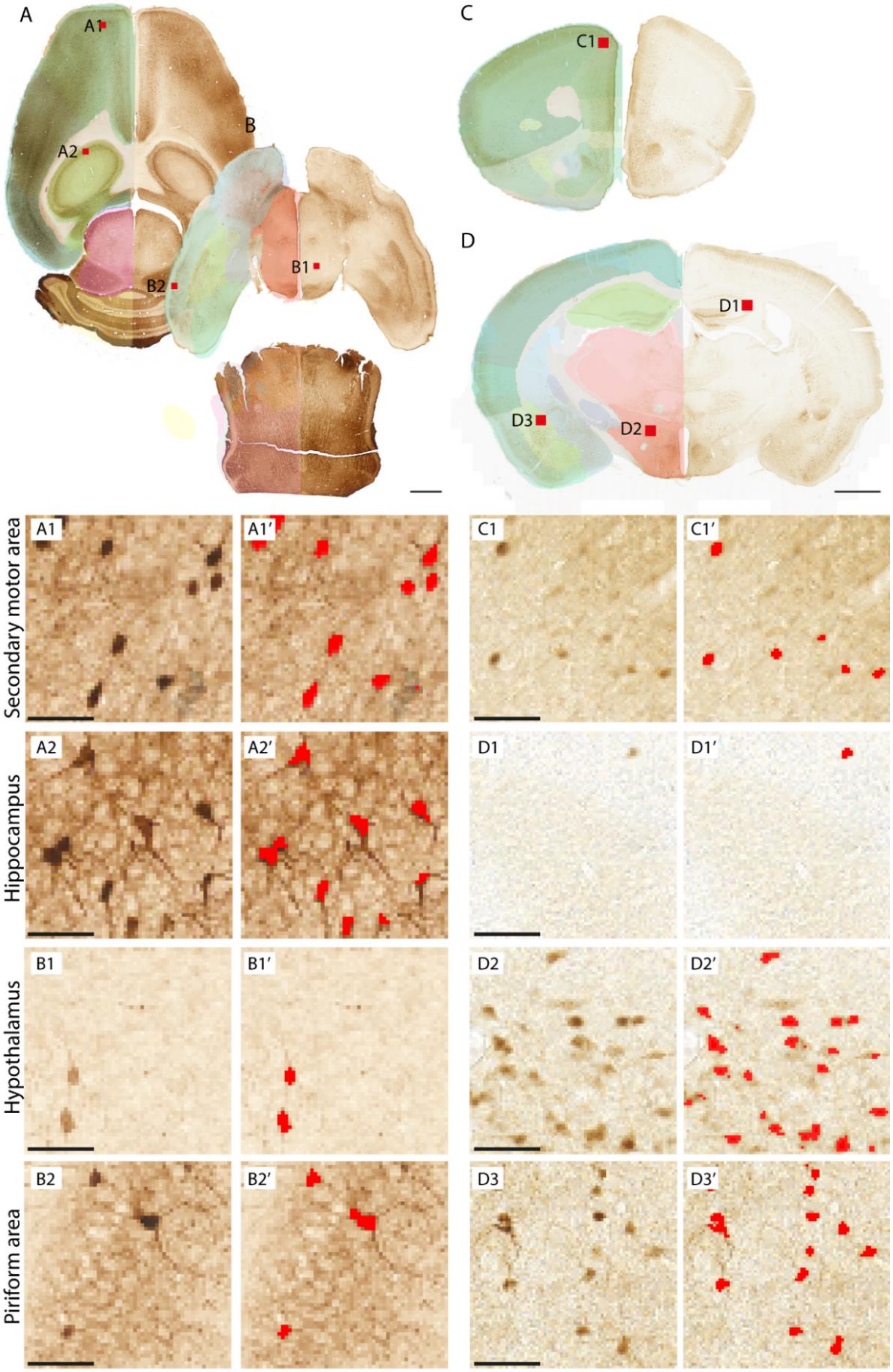

(A,B) show parvalbumin stained sections (from mouse 81265, sections 012 and 041, respectively), and (C,D) show calbindin stained sections (from mouse 6, sections 097 and 181, respectively). Atlas overlays are superimposed on one hemisphere. (A1-2, B1-2, C1, and D1-3) are enlarged images from selected regions as indicated in (A-D), showing regions secondary motor area (A1, C1), hippocampus (A2, D1), hypothalamus (A3, D2), and piriform area (A4, D3). Panels (A1'-2', B1'-2', C1' and D1'-3') show ilastik segmentations used for QUINT counts of labelled cells. Some lightly stained calbindin neurons (e.g. in hypothalamus) were not extracted by our classifier. Images are shown at the reduced resolution used for this analysis, high-resolution images are available from the shared datasets. Scale bars: upper panels, 1 mm; lower panels, 50 µm.

Supplemental table 1: List of calbindin and parvalbumin numbers and densities in the mouse brain (related to Figure 2)

**Volume:** region volume in mm^3 **Number:** bilateral total number estimate **Density:** estimated number per mm^3		Volume	Parvalbumin		Calbindin	
			Number	Density	Number	Density
**Isocortex (ISO)**						
FRP	Frontal pole, cerebral cortex	0.97	1 053 ± 148	1 081 ± 152	518 ± 214	532 ± 219
MOp	Primary motor area	12.19	15 446 ± 2 596	1 267 ± 213	10 727 ± 2 049	880 ± 168
MOs	Secondary motor area	13.10	16 519 ± 3 579	1 261 ± 273	10 254 ± 1 558	783 ± 119
Somatosensory areas (SS)						
SSp-n	Primary somatosensory area, nose	3.02	4 702 ± 204	1 557 ± 68	2 795 ± 513	926 ± 170
SSP-bfd	Primary somatosensory area, barrel field	6.29	10 518 ± 1 048	1 673 ± 167	6 164 ± 10 05	980 ± 160
SSP-ll	Primary somatosensory area, lower limb	2.35	4 272 ± 383	1 816 ± 163	3 711 ± 609	1 578 ± 259
SSP-m	Primary somatosensory area, mouth	6.21	7 289 ± 685	1 174 ± 110	4 854 ± 748	782 ± 120
SSP-ul	Primary somatosensory area, upper limb	3.77	6 659 ± 1 207	1 766 ± 320	4 566 ± 819	1 211 ± 217
SSP-tr	Primary somatosensory area, trunk	1.40	2 778 ± 425	1 982 ± 303	2 218 ± 253	1 582 ± 181
SSP-un	Primary somatosensory area, unassigned	1.26	2 478 ± 274	1 963 ± 217	1 537 ± 277	1 218 ± 220
SSs	Supplemental somatosensory area	9.01	13 299 ± 670	1 475 ± 74	10 155 ± 1 908	1 126 ± 212
Gustatory and visceral areas						
GU	Gustatory areas	1.77	2 116 ± 392	1 197 ± 222	3 343 ± 423	1 890 ± 239
VISC	Visceral areas	2.35	2 789 ± 287	1 187 ± 122	3 874 ± 521	1 649 ± 222
Auditory areas (AUD)						
AUDd	Dorsal auditory area	1.21	2 217 ± 302	1 825 ± 249	987 ± 133	813 ± 109
AUDp	Primary auditory area	2.15	4 173 ± 744	1 942 ± 346	1 920 ± 287	893 ± 133
AUDpo	Posterior auditory area	0.61	855 ± 163	1 397 ± 267	934 ± 136	1 526 ± 222
AUDv	Ventral auditory area	1.82	3 311 ± 607	1 821 ± 334	2 087 ± 271	1 148 ± 149
Visual areas (VIS)						
VISal	Anterolateral visual area	0.76	1 291 ± 183	1 703 ± 241	990 ± 104	1 306 ± 137
VISam	Anteromedial visual area	0.79	1 883 ± 393	2 376 ± 496	933 ± 104	1 178 ± 204
VISl	Lateral visual area	1.23	1978 ± 413	1 614 ± 337	1 772 ± 308	1 446 ± 251
VISp	Primary visual area	7.07	13 120 ± 2 514	1 855 ± 355	10 063 ± 1 333	1 423 ± 288
VISpl	Posterolateral visual area	0.79	1 190 ± 300	1 498 ± 378	1 150 ± 292	1 447 ± 367
VISpm	Posteromedial visual area	1.04	2 350 ± 381	2 241 ± 364	1 764 ± 285	1 682 ± 272
VISli	Laterointermediate area	0.49	688 ± 84	1 398 ± 170	791 ± 121	1 608 ± 246
VISpor	Postrhinal area	1.29	1 976 ± 458	1 541 ± 357	2 131 ± 165	1 662 ± 129
Anterior cingulate areas (ACA)						
ACAd	Anterior cingulate area, dorsal part	3.13	5 890 ± 1 570	1 881 ± 501	3 702 ± 570	1 182 ± 182
ACAv	Anterior cingulate area, ventral part	2.41	4 558 ± 706	1 893 ± 293	4 428 ± 505	1 838 ± 210
Prefrontal areas (PFA)						
PL	Prelimbic area	2.41	3 116 ± 551	1 293 ± 229	3 713 ± 322	1 541 ± 133
ILA	Infralimbic area	0.84	925 ± 190	1 095 ± 224	2 960 ± 211	3 506 ± 249
ORB	Orbital area	5.90	9 678 ± 1 632	1 640 ± 277	8 547 ± 1 376	1 448 ± 233
AGA	Agranular insular area	7.89	8 052 ± 1 290	1 020 ± 163	15 332 ± 1 448	1 942 ± 183
Retrosplenial areas (RSP)						
RSPagl	Retrosplenial area, lateral agranular part	2.35	3 822 ± 723	1 621 ± 307	3 563 ± 515	1 512 ± 218
RSPd	Retrosplenial area, dorsal part	3.80	5 142 ± 800	1 353 ± 211	3 052 ± 494	803 ± 130
RSPv	Retrosplenial area, ventral part	4.35	7 727 ± 398	1 777 ± 92	1 331 ± 239	306 ± 55

Other isocortical areas (ISOo)						
PTLp	Posterior parietal association areas	2.46	4 121 ± 704	1 677 ± 287	2 439 ± 94	993 ± 38
TEa	Temporal association areas	3.10	4 494 ± 957	1 449 ± 309	4 645 ± 425	1 497 ± 137
PERI	Perirhinal area	0.79	526 ± 130	666 ± 165	1 169 ± 169	1 481 ± 214
ECT	Ectorhinal area	1.71	1 823 ± 298	1 065 ± 174	2 795 ± 277	1 632 ± 162
**Olfactory areas (OLF)**						
MOB	Main olfactory bulb	21.49	10 714 ± 4 632	499 ± 216	18 784 ± 1 355	874 ± 63
AOB	Accessory olfactory bulb	0.65	65 ± 31	100 ± 48	107 ± 31	164 ± 48
AON	Accessory olfactory nucleus	4.87	2 093 ± 704	430 ± 145	9 332 ± 987	1 916 ± 203
TT	Taenia tecta	1.44	672 ± 235	466 ± 163	1 574 ± 144	1 092 ± 100
DP	Dorsal peduncular nucleus	0.50	754 ± 199	1 493 ± 394	1 677 ± 79	3 324 ± 156
PIR	Piriform area	11.57	6 108 ± 899	528 ± 78	17 379 ± 865	1 502 ± 75
NLOT	Nucleus of the lateral olfactory tract	0.32	113 ± 39	353 ± 122	563 ± 132	1 763 ± 414
COA	Cortical amygdalar area	3.27	1 392 ± 281	426 ± 86	7 589 ± 843	2 324 ± 258
PAA	Piriform-amygdalar area	1.19	237 ± 138	199 ± 116	1 660 ± 199	1 393 ± 167
TR	Postpiriform transition area	1.40	795 ± 178	566 ± 127	3 559 ± 417	2 534 ± 297
**Hippocampal formation (HPF)**						
Hippocampal region (HR)						
CA1	Field CA1	10.19	6 631 ± 1 221	651 ± 120	5 893 ± 1 257	578 ± 123
CA2	Field CA2	0.48	391 ± 61	823 ± 127	84 ± 19	177 ± 40
CA3	Field CA3	6.26	3 835 ± 950	612 ± 152	1 638 ± 110	262 ± 17
DG	Dentate gyrus	6.60	1 173 ± 372	178 ± 56	5 778 ± 896	876 ± 136
FC	Fasciola cinerea	0.06	61 ± 19	996 ± 304	1 ± 1	16 ± 16
IG	Induseum griseum	0.12	45 ± 15	355 ± 115	23 ± 7	183 ± 54
Retrohippocampal region (RHP)						
ENTl	Entorhinal area, lateral part	6.38	3 869 ± 478	607 ± 75	10 580 ± 1 257	1 659 ± 197
ENTm	Entorhinal area, medial part	5.02	8 384 ± 940	1 671 ± 187	4 962 ± 586	989 ± 117
PAR	Parasubiculum	0.93	2 439 ± 251	2 614 ± 269	658 ± 83	706 ± 89
POST	Postsubiculum	1.08	2 264 ± 397	2 088 ± 366	528 ± 112	487 ± 104
PRE	Presubiculum	0.92	2 314 ± 366	2 525 ± 400	479 ± 134	523 ± 146
SUB	Subiculum	2.10	4 347 ± 355	2 072 ± 169	909 ± 221	433 ± 106
ProS	Prosubiculum	1.33	1 550 ± 205	1 167 ± 155	924 ± 141	696 ± 106
HATA	Hippocampo-amygdalar transition area	0.42	441 ± 125	1 047 ± 296	1 061 ± 128	2 519 ± 303
APr	Area prostriata	0.32	432 ± 30	1 324 ± 91	279 ± 64	853 ± 263
**Cortical subplate (CTXsp)**						
CLA	Claustrum	0.55	397 ± 45	721 ± 82	859 ± 64	1 559 ± 116
EP	Endopiriform nucleus	2.79	1 330 ± 230	476 ± 82	6 186 ± 477	2 215 ± 171
LA	Lateral amygdalar nucleus	0.84	351 ± 65	418 ± 77	829 ± 130	986 ± 154
BLA	Basolateral amygdalar nucleus	1.90	1 151 ± 308	606 ± 162	4 486 ± 631	2 361 ± 332
BMA	Basomedial amygdalar nucleus	1.49	841 ± 245	567 ± 165	4 109 ± 681	2 766 ± 458
PA	Posterior amygdalar nucleus	0.97	1 304 ± 482	1 349 ± 499	5 554 ± 569	5 745 ± 589
**Cerebral nuclei (CNU)**						
Striatum (STR)						
CP	Caudoputamen	26.01	15 015 ± 519	577 ± 20	48 064 ± 7 665	1 847 ± 295
STRv	Striatum ventral region	4.83	1 578 ± 206	327 ± 43	10 027 ± 1 326	2 076 ± 275
OT	Olfactory tubercle	3.82	1 327 ± 283	347 ± 74	1 597 ± 182	418 ± 48
LSX	Lateral septal complex	3.56	444 ± 119	125 ± 33	11 005 ± 982	3 088 ± 276
sAMY	Striatum-like amygdalar nuclei	4.05	871 ± 200	215 ± 49	10 291 ± 1 188	2 540 ± 293
Pallidum (PAL)						
GPe	Globus pallidus, external segment	1.56	337 ± 220	216 ± 140	350 ± 38	223 ± 24
GPi	Globus pallidus, internal segment	0.42	108 ± 49	254 ± 115	24 ± 7	57 ± 16
PALv	Pallidum, ventral region	3.39	1 496 ± 298	441 ± 88	3 196 ± 462	942 ± 136
PALm	Pallidum, medial region	1.51	1 060 ± 359	701 ± 238	3 766 ± 712	2 492 ± 471
BST	Bed nuclei of the stria terminalis	1.34	216 ± 58	161 ± 43	5 832 ± 1 067	4 349 ± 796
**Thalamus (TH)**						
VENT	Ventral group of the dorsal thalamus	4.86	1 267 ± 315	261 ± 65	5 078 ± 1 282	1 045 ± 264
SPF	Subparafascicular nucleus	0.20	7 ± 3	32 ± 15	507 ± 97	2 474 ± 474
SPA	Subparafascicular area	0.13	4 ± 2	28 ± 14	658 ± 202	4 964 ± 1 526
PP	Peripeduncular area	0.06	6 ± 4	96 ± 68	389 ± 91	6 359 ± 1 482

GENd	Geniculate group, dorsal thalamus	1.42	58 ± 26	41 ± 18	893 ± 241	628 ± 170
LAT	Lateral group of the dorsal thalamus	3.08	323 ± 98	105 ± 32	3 540 ± 860	1 151 ± 279
ATN	Anterior group of the dorsal thalamus	2.16	304 ± 70	141 ± 33	1 084 ± 314	502 ± 146
MED	Medial group of the dorsal thalamus	2.08	247 ± 33	123 ± 16	5 844 ± 1 328	2 897 ± 658
MTN	Midline group of the dorsal thalamus	1.19	121 ± 44	102 ± 37	5 438 ± 976	4 585 ± 823
ILM	Intralaminar nuclei of the dorsal thalamus	1.60	78 ± 29	49 ± 18	3 880 ± 932	2 424 ± 582
RT	Reticular nucleus of the thalamus	-	-	-	416 ± 70	287 ± 48
GENv	Geniculate group, ventral thalamus	0.52	282 ± 118	539 ± 225	405 ± 71	773 ± 136
EPI	Epithalamus	0.66	124 ± 58	186 ± 87	1 920 ± 285	2 888 ± 429
**Hypothalamus (HY)**						
PVZ	Periventricular zone	0.78	62 ± 28	79 ± 36	2 046 ± 240	2 614 ± 307
PVR	Periventricular region	2.04	580 ± 126	283 ± 61	5 859 ± 815	2 864 ± 398
AHN	Anterior hypothalamic nucleus	0.71	274 ± 103	385 ± 145	3 881 ± 592	5 462 ± 833
MBO	Mammillary body	1.02	902 ± 175	888 ± 172	3 779 ± 578	3 720 ± 569
MPN	Medial preoptic nucleus	0.41	47 ± 16	115 ± 40	3 253 ± 396	8 003 ± 975
PM	Premammillary nuclei	0.33	113 ± 37	344 ± 113	721 ±234	2 202 ± 716
PVH	Paraventricular hypothalamic nucleus, descending division	0.13	13 ± 8	101 ± 64	542 ± 29	4 150 ± 219
VMH	Ventromedial hypothalamic nucleus	0.55	85 ± 19	154 ± 35	3 460 ± 404	6 298 ± 736
PH	Posterior hypothalamic nucleus	0.71	65 ± 18	92 ± 26	893 ± 255	1 263 ± 360
LZ	Hypothalamic lateral zone	5.69	2 017 ± 620	355 ± 109	8 049 ± 1 250	1 416 ± 220
ME	Median eminence	0.09	0 ± 0	0 ± 0	6 ± 5	74 ± 60
**Midbrain (MB)**						
Midbrain, sensory related (MBsen)						
SCs	Superior colliculus, sensory related	2.16	3 813 ± 554	1 768 ± 257	7 213 ± 928	3 345 ± 430
IC	Inferior colliculus	4.44	6 011 ± 1 424	1 352 ± 320	450 ± 65	101 ± 15
NB	Nucleus of the brachium of the inferior colliculus	0.09	67 ± 19	766 ± 214	32 ± 11	359 ± 129
SAG	Nucleus sagulum	0.10	66 ± 38	671 ± 391	15 ± 4	148 ± 43
PBG	Parabigeminal nucleus	0.04	26 ± 11	572 ± 254	3 ± 2	45 ± 45
MEV	Midbrain trigeminal nucleus	0.01	2 ± 1	231 ± 148	15 ± 3	1 585 ± 289
SCO	Subcommissural organ	0.01	8 ± 5	556 ± 313	3 ± 2	208 ± 138
SN	Substantia nigra	1.74	342 ± 138	196 ± 79	132 ± 39	76 ± 23
Midbrain, motor related (MBmot)						
VTA	Ventral tegmental area	0.43	146 ± 60	341 ± 140	1 860 ± 392	4 339 ± 914
PN	Paranigral nucleus	0.02	2 ± 1	101 ± 64	64 ± 13	2 951 ± 620
MRN	Midbrain reticular nucleus	5.28	2 449 ± 733	464 ± 139	901 ± 241	171 ± 46
SCm	Superior colliculus, motor related	5.65	3 981 ± 1 345	704 ± 238	2 716 ± 836	480 ± 148
PAG	Periaqueductal grey	4.69	833 ± 352	178 ± 75	5 962 ± 1 231	1 270 ± 262
PRT	Pretectal region	2.08	1 380 ± 434	663 ± 209	578 ± 119	278 ± 57
MBm-o	Midbrain, motor related, other	1.60	822 ± 152	517 ± 96	296 ± 55	186 ± 35
Midbrain, behavioral state related (MBsta)						
PPN	Pedunculopontine nucleus	0.89	332 ± 98	372 ± 110	461 ± 133	516 ± 149
RAmb	Midbrain raphe nuclei	0.73	345 ± 127	471 ± 173	1 373 ± 272	1 872 ± 371
**Pons (PE)**						
P-sen	Pons, sensory related	3.85	5 099 ± 483	1 324 ± 126	3 128 ± 691	812 ± 179
P-mot	Pons, motor related	8.16	2 478 ± 1 092	304 ± 30	985 ± 156	121 ± 19
P-sat	Pons, behavioral state related	1.10	1 015 ± 285	923 ± 259	871 ± 248	792 ± 226
**Medulla (MY)**						
MY-ua	Medulla, unassigned	5.32	2 545 ± 483	478 ± 91	1 032 ± 409	194 ± 77
MY-sen	Medulla, sensory related	7.83	13 232 ± 1 092	1 690 ± 140	4 821 ± 2 155	616 ± 275
MY-mot	Medulla, motor related	19.75	11 369 ± 1 141	576 ± 58	3 493 ± 1 090	177 ± 55
MY-sat	Medulla, behavioral state related	0.22	55 ± 14	245 ± 63	30 ± 10	134 ± 46

Supplemental Table 2: List of mouse parvalbumin literature sources (related to Figure 5)

Mouse parvalbumin neuron densities								
**Source**	**PMID**	**Strain**	**Brain region**	**# of animals**	**Counting method**	**Section thickness**	**Density (mm^3)**	**Antibody**
Grünewald et al., 2017	29135436	129/Sv	Amygdala (whole region)	14	Direct count	30	2 649	RRID: AB_2631173
Fasulo et al., 2017	28232789	Other	Basolateral amygdala	4	Stereology	40	1 400	No info
Jinno and Kosaka, 2006	16930755	C57BL/6J	CA1	4	Stereology	50	1 405	Kägi et al. (1987)
Neddens and Buonanno, 2009	19655320	C57BL/6	CA1	4	Direct count	50	1 158	rabbit polyclonal anti pv, Swant
Pitts et al., 2013	23880772	C57BL/6J	CA1 of dorsal hippocampus	6	Stereology	40	1 480	RRID:AB_10000344
Pitts et al., 2013	23880772	C57BL/6J	CA2/3 of dorsal hippocampus	6	Stereology	40	2 171	RRID:AB_10000344
Jinno and Kosaka, 2006	16930755	C57BL/6J	CA3	4	Stereology	50	1 535	Kägi et al. (1987)
Neddens and Buonanno, 2009	19655320	C57BL/6	CA3	4	Direct count	50	1 127	rabbit polyclonal anti pv, Swant
Grünewald et al., 2017	29135436	129/Sv	CA3	14	Stereology	30	5 796	RRID: AB_2631173
Filice et al., 2016*	26819149	C57BL/6J	Caudoputamen	5	Stereology		897	RRID:AB_10000344
Lauber et al., 2018*	30116174	C57BL/6J	Caudoputamen	6	Stereology		819	RRID:AB_10000344
Lauber et al., 2016*	28066177	C57BL/6J	Caudoputamen	5	Stereology		817	RRID:AB_10000344
Andsberg et al., 2001*	11358448	C57BL/6J	Caudoputamen	7	Stereology	40	864	polyclonal rabbit anti pv
Smith et al., 2008	17988653	Other	Caudoputamen	3	Stereology	50	3 522	No info
Yalcin-Cakmakli et al., 2018*	29997483	C57BL/6J x DBA/2J	Caudoputamen	3	Stereology	30	953	RRID:AB_2631173
Song et al., 2013*	23336980	C57BL/6J	Caudoputamen	12	Stereology	40	612	No info
Filice et al., 2016*	26819149	C57BL/6J	Caudoputamen	5	Stereology		714	RRID:AB_10000344
Ransome and Turnley, 2005	15837129	C57BL/6	Caudoputamen	3	Stereology	30	1 060	mouse anti-parvalbumin, Chemicon
Förster, 2008	**	C57BL/6J	Caudoputamen	6	Stereology	25	1 430	PARV-19, Sigma
Fasulo et al., 2017	28232789	Other	Dentate gyrus	4	Stereology	40	400	No info
Fasulo et al., 2017	28232789	Other	Dentate gyrus	4	Stereology	40	1 200	No info
Fasulo et al., 2017	28232789	Other	Dentate gyrus	4	Stereology	40	1 100	No info
Jinno and Kosaka, 2006	16930755	C57BL/6J	Dentate gyrus	4	Stereology	50	650	Kägi et al. (1987)
Neddens and Buonanno, 2009	19655320	C57BL/6	Dentate gyrus	4	Direct count	50	745	rabbit polyclonal anti pv, Swant
Pitts et al., 2013	23880772	C57BL/6J	Dentate gyrus of dorsal hippocampus	6	Stereology	40	493	RRID:AB_10000344
Smith et al., 2008*	17988653	Other	Globus pallidus	3	Stereology	50	40 194	No info
Pitts et al., 2013	23880772	C57BL/6J	Inferior colliculus	6	Stereology	40	4 836	RRID:AB_10000344
Moreno-Gonzalez et al., 2009	19661615	C57BL/6	Lateral entorhinal area	5	Stereology	40	4 300	rabbit polyclonal anti pv, Swant
Parrish-Aungst et al., 2007	17311323	C57BL/6J	Main olfactory bulb	4	Stereology	25	2 324	RRID:AB_10000343
Pirone et al., 2018	30369876	Other	Medial prefrontal cortex (infralimbic)	3	Direct count	20	2 052	RRID: AB_2631173
Pirone et al., 2018	30369876	Other	Medial prefrontal cortex (prelimbic)	3	Direct count	20	3 167	RRID: AB_2631173
Pitts et al., 2013	23880772	C57BL/6J	Medial septum	6	Stereology	40	1 678	RRID:AB_10000344
Smith et al., 2008*	17988653	Other	Nucleus accumbens	3	Stereology	50	2 557	No info

Source	PMID	Strain	Brain region	# of animals	Counting method	Section thickness	Density (mm^3)	Antibody
Sanchez-Mejias et al., 2020	31491047	C57BL/6J	Perirhinal area 35	3	Stereology	40	10 765	No info
Sanchez-Mejias et al., 2020	31491047	C57BL/6J	Perirhinal area 36	3	Stereology	40	10 974	No info
Ransome and Turnley, 2005	15837129	C57BL/6	Somatosensory cortex	3	Stereology	30	4 580	mouse anti-parvalbumin, Chemicon
Pitts et al., 2013	23880772	C57BL/6J	Somatosensory cortex	6	Stereology	40	5 329	RRID:AB_10000344
Neddens and Buonanno, 2009	19655320	C57BL/6	Subiculum	4	Direct count	50	2 048	rabbit polyclonal anti pv, Swant
Trujillo-Estrada et al., 2014	24927710	C57BL/6	Subiculum	5	Stereology	40	10 721	rabbit polyclonal anti pv, Swant

* These densities were obtained by dividing total number estimates reported in sources by the volume of the region (as defined by the Allen Mouse brain Common Coordinate Framework, version 3, 2017 edition; see main text for details).

** Thesis, no PMID available. Available from: http://citeseerx.ist.psu.edu/viewdoc/download?doi=10.1.1.427.6474&rep=rep1&type=pdf

Supplemental Table 3: List of mouse calbindin literature sources (related to Figure 2)

Mouse calbindin neuron densities								
Source	PMID	Strain	Brain region	# of animals	Counting method	Section thickness	Density per mm^3	Antibody
Grünewald et al., 2017	29135436	129/Sv	Amygdala (whole region)	12	Direct count	30	5 005	RRID: AB_10000340
Grünewald et al., 2017	29135436	129/Sv	CA3	14	Stereology	30	945	RRID: AB_10000340
Jinno and Kosaka, 2006	16930755	C57BL/6J	CA1	4	Stereology	50	1 555	Pinol et al. (1990)
Jinno and Kosaka, 2006	16930755	C57BL/6J	CA3	4	Stereology	50	1 590	Pinol et al. (1990)
Jinno and Kosaka, 2006	16930755	C57BL/6J	DG	4	Stereology	50	210	Pinol et al. (1990)
Parrish-Aungst et al., 2007	17311323	C57BL/6J	Main olfactory bulb	4	Stereology	25	10 916	RRID:AB_2721225

Supplemental Table 4: List of rat parvalbumin literature sources (related to Figure 2)

Rat parvalbumin neuron densities								
**Source**		**Strain**	**Brain region**	**# of animals**	**Counting method**	**Section thickness**	**Density (mm^3)**	**Antibody**
Wang et al., 2008	18059437	Sprague-Dawley	CA1		Direct count	50	1 600	RRID:AB_477329
Bezaire and Soltesz, 2013	23674373	-	CA1	-	See legend*	-	-	-
Aika et al., 1994	7925807	Wistar	CA1, dorsal	5	Stereology	0,5	1 100	Polyclonal rabbit anti PV antibody
Wang et al., 2008	18059437	Sprague-Dawley	Caudoputamen		Direct count	50	846	RRID:AB_477329
Kaalund et al., 2013	23083323	Lister Hooded	Dentate gyrus		Stereology	60	2 128	No info
Megahed et al., 2015	25620912	Sprague-Dawley	Dentate gyrus	6	Stereology	30	2 125	No info
Shiraki et al., 2016	27553673	Sprague-Dawley	Dentate gyrus hilus	10	Direct count	3	1 461	PARV-19 Millipore
Kaalund et al., 2013	23083323	Lister Hooded	Hippocampus CA		Stereology	60	2 994	No info
Megahed et al., 2015	25620912	Sprague-Dawley	Hippocampus CA	6	Stereology	30	3 064	No info
Barinka et al., 2012	22221733	Wistar	Perirhinal area 35	6	Stereology		3 805	Monoclonal mouse anti PV
Barinka et al., 2012	22221733	Wistar	Perirhinal area 36	6	Stereology		4 336	Monoclonal mouse anti PV

***** Bezaire & Soltesz (2013) used a combination of literature derived data and calculations to arrive at an estimate of parvalbumin neurons in the rat CA1 (10010 parvalbumin positive neurons unilaterally). Our estimate is 9 198 parvalbumin positive neurons unilaterally in rat CA1.

Transparent methods

EXPERIMENTAL MODEL AND SUBJECT DETAILS

Experimental procedures involving animals were approved by the Norwegian Food Safety Authority and carried out in accordance with the European Union and International legislation for the use of animal subjects. Four adult (6 months old) male PVCre X Rosa26eYFP mice (RRID:IMSR_JAX:008069 and RRID:IMSR_JAX:007903, respectively, crossed locally) and four adult female Sprague-Dawley rats (3 months old, Charles River, Sulzfeld/Kisslegg, Germany) housed at the Kavli Institute, Norwegian Institute of Science and Technology (NTNU), Norway were used for parvalbumin immunohistochemistry. Six adult C57Bl6/J mice (four females and two males, four months old, females from Janvier Labs and males from Taconic), housed at the Institute for Basic Medical Sciences, University of Oslo, Norway, were used for calbindin immunohistochemistry. Animals were housed according to the recommendations by FELASA under pathogen free conditions, with the exception of the presence of Entamoeba muris detected by PCR in fecal samples for the rats. Up to two rats and five mice of the same sex were group-housed in transparent, semi-enriched cages with 12:12 reversed day / night cycles and ad libitum access to food and water.

DATA ACQUISITION

Immunohistochemistry

For parvalbumin immunohistochemistry, animals were deeply anesthetised with sodium pentobarbital (50 mg/kg body weight) and transcardially perfused with Ringer solution containing 4 % paraformaldehyde (PFA). Brains were postfixed in PFA for approximately 24 hours and cryoprotected in a solution of 2 % DMSO and 20 % glycerol in phosphate buffer (PB). Horizontal sections were cut at 30 µm (mouse brains) or 40 µm (rat brains) on a freezing microtome. For calbindin immunohistochemistry, mice were anesthetized with isofluorane, given an overdose of Zoletil mixture, and transcardially perfused with NAPI followed by 4 % PFA. The brains were postfixed overnight in the same fixative, and transferred to 0.4 % PFA for storage. Prior to cutting, the brains were cryoprotected by immersion in 10, 20 and 30 % sucrose until they sank. The right hemisphere was marked by making shallow cut in the cortical surface. Brains were coronally divided at the level of the dorsal hippocampus before coronal sections of 40 µm were cut using a freezing microtome. For all experiments, free-floating sections were used for immunohistochemistry according to the avidin-biotin peroxidase method, using 3,3'-diaminobenzidine (DAB) as the chromogen (every fourth for parvalbumin immunohistochemistry, and every sixth for calbindin). All subsequent steps were performed at room temperature, unless otherwise specified.

Parvalbumin immunohistochemistry. Sections were rinsed with PB before blocking endogenous peroxidase activity by incubation in a solution of 3 % H_2O_2 and 10 % methanol in PB. Sections were rinsed again with PB, but subsequent washes were done using Tris buffered saline with 0.5% triton (TBS-TX). The sections were pre-incubated for 1 hour in 5 % normal goat serum in TBS-TX before overnight incubation at 4°C with the primary antibodies (diluted 1:4000 in TBS-TX). A monoclonal mouse anti-parvalbumin antibody (RRID:AB_477329) was used for the rat brain sections, and a monoclonal rabbit anti parvalbumin (RRID:AB_2631173) for the mouse brain sections. After rinsing, sections were incubated overnight at 4°C with polyclonal, biotinylated secondary antibodies: a goat anti mouse (RRID:AB_258604) antibody for the rat brain sections and a goat anti rabbit (RRID:AB_258649) antibody for the mouse brain sections. Sections were rinsed and then incubated for 1.5 hour with the VectaStain ABC HRP kit, according to manufacturer's instructions. After rinsing again with TBS-TX, the sections were rinsed twice with Tris-HCl before incubation with the DAB solution. The solution was made by adding a 10 mg DAB tablet to 20 mL Tris-HCl, and the tablet was dissolved by placing the solution on a heated stirrer for 2 hours. Afterwards, H_20_2 was added to the solution, which was then placed in the freezer for 20 minutes to slow down the reaction with the sections. The sections were incubated with the DAB solution for approximately 3 minutes and then rinsed with Tris-HCl. The sections were mounted on Superfrost microscope slides with Tris-HCl, and then dried and coverslipped using Entellan (Merck Millipore, Darmstadt, Germany).

Calbindin immunohistochemistry. Sections were rinsed with PBS three times. Endogeneous peroxidase activity was blocked by incubating sections with 3% H_2O_2 for five minutes. After three brief rinses in PBS, sections were incubated in blocking solution (3% normal donkey serum, 1% BSA and 0.1% triton X in PBS) for 1 hour. Sections were incubated in the primary antibody (monoclonal mouse anti calbindin, Swant 300, RRID:AB_10000347, lot no. 07F) overnight at 4°C. The antibody is specific to calbindin-D28k and does not bind to calretinin or other known calcium bindin proteins (manufacturer's description). Control sections processed without the primary antibody did not show specific labelling, and are shared together with the data sets. The next day, sections were washed in PBS before incubation with the secondary antibody (sheep anti mouse, GE Healthcare RPN1001V) for one hour. After rinsing, sections were incubated with ABC kit for 30 minutes, and rinsed again with PBS. Sections were then reacted with DAB and H_2O_2 (kit from Abcam, used according to manufacturer's instructions) for five minutes. The reaction was stopped with distilled water. Sections were mounted from PBS onto gelatinized microscope slides, dehydrated through an ascending series of ethanol (70, 90 and 100 % for two minutes each) followed by two times two minutes in xylene, dried and coverslipped with Entellan.

Scanning and image pre-processing

Sections were scanned with a 20× objective using a Zeiss Axioscan Z1 scanner (Carl Zeiss MicroImaging, Jena, Germany) and loss less TIFF files exported from the Zen software (RRID:SCR_013672). The TIFF images were renamed to reflect their serial order, and sections that had been horizontally flipped during mounting were mirrored. Images were downscaled to the lowest resolution giving satisfactory segmentation of cell bodies with the ilastik software (15 % and 10 % of their original width for parvalbumin and calbindin images, respectively). Mirroring, renaming and resizing steps were performed using the Transform function of the Nutil software (v.0.4.01, RRID:SCR_017183; Groeneboom et al., 2020). Distortions and dislocation of individual tissue pieces introduced during processing and mounting were corrected using Adobe Photoshop. We moved and mirrored image parts to reconstruct normal section appearance. For parvalbumin sections, such corrections were applied to the original TIFF images. However, the calbindin TIFF images were too large to be opened in Adobe Photoshop or similar software, and correction of section parts were therefore only applied to the downscaled images.

DATA ANALYSIS

We used the QuickNII-ilastik-Nutil (QUINT) workflow to extract and quantify labelled cells from microscopic images of calbindin- and parvalbumin stained sections (Yates et al., 2019). Through this workflow, images are spatially registered to atlas using QuickNII; objects-of-interest (in our case, cells) are segmented from the images using ilastik; and the segmented objects are quantified using Nutil. To extrapolate estimates for total numbers and volumetric densities, we further post-processed the QUINT results. Lastly, we performed a comparative analysis of parvalbumin neurons in the rat and mouse hippocampal region. The detailed methods of each step of the QUINT workflow, the post-processing of the results, and the comparative analysis is described below.

Atlas registration

Serial section images were spatially registered to common 3-D brain reference atlases using the QuickNII software v2.2 (RRID:SCR_016854; Puchades et al., 2019), bundled with the Allen Mouse Common Coordinate Framework, version 3 of the template, 2017 edition of the delineations (Wang et al., 2020; here referred to as CCFv3-2017) and the Waxholm Space atlas of the Sprague-Dawley rat brain, version 1.01 of the template and version 2 of the delineations (Papp et al., 2014; Kjonigsen et al., 2015; here referred to as WHSv2). First, section images were registered using QuickNII to identify section positions and deviations from the standard planes. For coronally oriented sections, the anatomically distinct landmarks were the

genu of the corpus callosum, the crossing of the anterior commissure, and the rostral appearance of structures such as nucleus accumbens, caudoputamen, and dorsal hippocampus. The superior and inferior colliculus, as well as the inferior olive, provided important anatomical landmarks in more posteriorly located sections. For horizontally oriented sections, the registration was primarily based on the anterior commissure, the dorsal appearance of the hippocampus and caudoputamen, the olfactory bulb and the piriform cortex. A second researcher verified all registration results. The affine registration with QuickNII yielded custom atlas plates matching each section image. To further optimize registration, the custom atlas images were non-linearly transformed using the software tool VisuAlign v0.8 (RRID:SCR_017978). We first focused on fitting the template to the outer edges of the section, and secondly adjusted it to refine the fit of major landmarks situated deeper in the brain (e.g. striatum, globus pallidus, hippocampus). The high-resolution microscopic images with their custom atlas overlay images are organized and disseminated to an interactive web-microscopy viewer via the Navigator N3 data system at the University of Oslo. A forerunner of this system is described by Moene et al. (2007).

Image segmentation

Pixel classification with ilastik. Images were segmented using the pixel classification pipeline in ilastik (RRID:SCR_015246; v.1.3.3), which allows classification of features on a pixel level on a scale up to 10 by 10 pixels. We assigned two label classes termed “cell” and “background”. During segmentation, we placed labels throughout different regions and in all the training sections until the segmentation was deemed satisfactory. We trained one classifier per image series (i.e. one per animal). For each series, we used every fifth image for training and then applied the classifier to all images using the Batch Processing function in ilastik.

Removal of artefacts and incorrectly segmented objects. Overall, the classifiers produced by the pixel classification pipeline distinguished objects of interest from background with relatively high accuracy. Still, some artefacts and incorrectly segmented objects occurred. The ilastik software includes an object classification pipeline that allows for distinguishing artefacts, which is especially useful when these are very different from the objects of interest. However, in our material incorrectly segmented objects (e.g. neurites) resembled the objects of interest in size and shape. Therefore, we visually inspected all segmentation images to identify incorrectly segmented objects or artefacts, and removed these manually using NIH ImageJ. Most artefacts segmented as “cell” were seen around the edges of the sections and in relation to blood vessels and ventricles. Example images, segmentations and atlas overlays are shown in Supplemental Figure 4.

Quantification of segmented objects

We used Nutil Quantifier (v.0.4.02) to combine customized atlas maps from QuickNII (refined and exported using VisuAlign) with segmented images from ilastik. The pixel size cut-off function was used to exclude objects representing fragments or small artefacts; a cut-off of 4 pixels was chosen for both parvalbumin and calbindin datasets, based on visual inspection of segmented images.

The regions in the CCFv3-2017 are more fine-grained than the WHSv2. To achieve better correspondence with the granularity of the rat brain atlas, small regions and individual cellular layers in CCFv3-2017 were grouped into more coarse areas (e.g. primary motor cortex, CA1 of the hippocampus) using the “custom regions” feature of Nutil Quantifier. This reduction of granularity of atlas delineations was also motivated by the need to compensate for inaccuracies in the spatial registration of images to the common 3D atlas, which primarily impacts the precision of registration of small brain regions. Excel sheets indicating the organization of custom atlas regions are provided with the derived datasets and can be re-used in new analyses with Nutil Quantifier. Post-processing of numerical data (correction and extrapolation to whole regions and volumetric densities, described below) from mouse brains was performed on the numbers from these

aggregated custom regions. The numbers per individual region in the CCFv3-2017 were also extracted with Nutil Quantifier and included in the derived data files. The custom regions used in the present analyses are given in Figure 1.

Post-processing of Nutil Quantifier results

The output reports from Nutil contain the total number of segmented objects counted in every sampled section through each atlas region, given as counts per region, section, and region for the whole brain. These numbers represent all objects counted in all the images investigated. Additionally, damaged or missing parts of individual sections might influence results. To correct for the tissue sampling and missing tissue parts, we implemented a series of post-processing steps to extrapolate Nutil Quantifier numbers to reflect densities and total number estimates. First, we used the sectional reports to compile numbers per section for each region in a new spreadsheet. For all series, we manually inspected the quality and completeness of each section image. Whenever a part of the brain was damaged or missing in a section, or staining quality was deemed suboptimal, we identified which region(s) of the brain this affected. For every such instance, we replaced the Nutil Quantifier results according to the following rules. If a section showed damage or suboptimal staining on one side of the brain, we used the intact side as a reference and multiplied the number by two to get a bilateral estimate for the section. If a region was damaged or sub-optimally stained on both sides, we used an average of numbers obtained from the adjacent neighbouring sections. The adjusted section-by-section numbers were summed per region of interest in a second spreadsheet. On average, 20 % of the sections in a series needed some form of correction, typically meaning that numbers for one or a few regions were corrected as described above. Exclusion of whole sections from the analysis were very rarely necessary, and never for more than 4 % of all the sections within a series. Details on whether corrections were made (and if so, which ones) are provided as metadata with each of the datasets.

Secondly, to adjust for double-counting in volumetric extrapolation, we calculated an estimate of total numbers of cells in each region by using Abercrombie's formula (Abercrombie, 1946):

$$N = \frac{n \times T}{T + D}$$

Where N is the cell number, n is the number of counted profiles, T is the thickness of the sections, and D is the mean diameter of the profiles. The mean cell diameter was calculated by multiplying the average object area for all cells (generated by Nutil Quantifier) by the pixel scale of the images, and using this area (A) to calculate the diameter (D):

$$D = 2 \times \frac{A}{\pi}$$

The pixel scales for the section images used in ilastik were 8.6 pixels per μm^2 for rat and mouse parvalbumin section images, and 4.8 pixels per μm^2 for the mouse calbindin section images. The estimated diameter of the cells used in our calculations were 11.3 and 12.7 µm for mouse and rat parvalbumin neurons, respectively, and 7.9 µm for mouse calbindin neurons. Lastly, we multiplied the resulting number with the section sampling fraction (4 for parvalbumin datasets, 6 for calbindin datasets) to estimate the total number of cells per region.

To estimate volumetric densities, we divided the total number of cells for each region with the volumes of each atlas region, exported from the ITK-snap software using the "Volumes and statistics" function. The .xlsx files included in the derived datasets, published on EBRAINS.eu, contain all the raw and corrected numbers, as well as the region volumes used for calculations. The volumetric densities estimated here were compared against those reported by Kim et al. (2017) and others in the literature (see section on "Comparison of findings with data from previous reports" below).

Comparative analysis of parvalbumin neurons

To compare the total number and density of parvalbumin neurons in the mouse and rat hippocampal region, hippocampal data from regions in the CCFv3-2017 were grouped to correspond with rat brain regions defined in the WHSv2 rat brain atlas. Most areas of the hippocampal region were highly comparable between the atlases, in terms of nomenclature, anatomical position and shape of the regions (Figure 6). The regions of Ammon's horn and the dentate gyrus were highly similar. The prosubiculum of the CCFv3-2017, intercalated between the subiculum and the CA1, was grouped as part of the subiculum, while the postsubiculum was included in the presubiculum, as this term is commonly used to refer to the dorsal presubiculum (Taube, 2007; Taube et al., 1990). The perirhinal and ectorhinal cortices in the CCFv3-2017 was considered to correspond to WHSv2 perirhinal area 35 and 36, respectively. Lastly, the postrhinal cortex (POR), was present in both the CCF and WHSv2, but with a much narrower dorsoventral extent in the CCFv3-2017 than the WHSv2. In the WHSv2, the POR lines the caudal pole of the lateral and medial entorhinal cortex dorsally; in the CCFv3-2017, however, the POR does not extend to the most caudal part of the cortex. At this level, a cortical region termed the posterolateral visual area is included in the CCFv3-2017. Anatomically, the combined postrhinal and posterolateral visual area in the CCFv3-2017 resemble the POR in the WHSv2. We therefore included both of these in the postrhinal cortex for our comparative analysis. Figure 6 shows hippocampal regions in the two atlases and the mapping of CCFv3-2017 to WHSv2 regions.

Spatial distribution analysis. To explore and compare possible topographical distribution gradients of parvalbumin neurons in the mouse and rat parahippocampal region, we averaged the section-by-section density of parvalbumin neurons across animals and assessed whether densities of parvalbumin stained neurons changed along the dorsoventral axis of each subregion. To get numbers that could be compared for each level, we calculated volumetric densities per section as follows. First, we calculated corrected numbers of cells for each atlas region per section by applying Abercrombie's formula to the section-wise numbers. Then, for each atlas region, we multiplied the region area in pixels (extracted from Nutil Quantifier reports) by the pixel scale (see above). For each section, we then divided the corrected number of cells per region by the region area, and divided this by the section thickness (Keller et al., 2018) to get the volumetric density:

$$\mathrm{vN} = \frac{n}{T}$$

Where vN is the volumetric density, n is the corrected two-dimensional cell count, and T is the thickness of the section. This gave the density per μm^3; lastly, to get numbers per mm^3, we therefore multiplied these by 10^9.

We next sorted sections according to their dorsoventral level for each animal, and averaged the density of neurons per level across animals. Each subregion was then represented in a spreadsheet as a row containing the average parvalbumin neuron density for each dorsoventral level (one column per level). We applied conditional formatting to each row so each cell was colour-coded in a gradient according to its value, with the lowest number for each row coded light yellow and the highest number coded dark orange. These colour-coded rows were inspected for cases of clear dorsoventral gradients and copied to Adobe Illustrator to be shown in figures (see Results).

METHOD VALIDATION

Comparison of findings with previous reports

Kim and colleagues (2017) mapped parvalbumin interneurons across the whole mouse brain using transgenic mice and image segmentation based on convolutional neural networks. They shared all the total number and density estimates through their publication, which we downloaded and used for comparison. They used a custom-built three-dimensional version of the original (two-dimensional) Allen Reference Atlas of the mouse brain (Dong, 2008). The nomenclature used for this version is similar, but not identical, to the one in the version we used in our analysis (CCFv3-2017). We therefore mapped the results found in their files to the custom regions used in our analysis (see above). Kim et al. (2017) reported values for both male and female mice, and reported that several subcortical structures are sexually dimorphic. Given this finding, and the fact that our mouse parvalbumin data were obtained from males, we used only the results from their male subjects (n = 5) for comparison to our results (n = 4).

We also queried the literature to find estimates of the same cell types as those quantified here. To this end, we searched for 1) the RRIDs and catalogue numbers of the antibodies used in our study (using Google Scholar) and 2) articles that mentioned both stereology and "parvalbumin" or "calbindin" in the title or abstract (using PubMed). For each search, all results were screened manually and included if they presented relevant data (total number or density estimate of parvalbumin or calbindin cells in rats or mice).

The following searches were performed:

1. Google Scholar search for RRID:AB_2631173. Performed 14.04.2020; 31 results, 2 included.

2. Google Scholar search for RRID:AB_477329. Performed 20.04.2020; 81 results, none included.

3. Google Scholar search for RRID:AB_10000347. Performed 21.04.2020; 87 results, none included.

4. Google Scholar search for ((rat) OR (mouse)) AND (Swant PV27) AND (stereology OR stereological). Performed 14.04.2020; 18 results, 1 included.

5. Google Scholar search for ((rat) OR (mouse)) AND (PARV-19) AND (stereology OR stereological). Performed 20.04.2020; 61 results, 2 included.

6. Google Scholar search for ((rat) OR (mouse)) AND ("Swant 300") AND (stereology OR stereological). Performed 21.04.2020; 21 results, none included.

7. PubMed (via Ovid Medline) search for: (rat or mouse or rodent).tw,kf AND stereolog*.tw,kf. AND parvalbumin.tw,kf. Performed 14.04.2020; 59 results, 14 included.

8. PubMed (via Ovid Medline) search for: (rat or mouse or rodent).tw,kf AND stereolog*.tw,kf. AND calbindin*.tw,kf. Performed 21.04.2020; 38 results, 3 included

We supplemented this search with references from a recent review of number estimates in the mouse brain (Keller et al., 2018) and from our previously published database of literature-derived quantitative estimates in the basal ganglia (Bjerke et al., 2019, 2020)

Validity of segmentations

The results obtained with the QUINT workflow were benchmarked against prior manual counts from both parvalbumin and calbindin stained material that were used as a guide during the segmentation process. Manual counting was performed on a subset of the material by annotating each labelled cell in Adobe Photoshop (RRID:SCR_014199). The annotated cells were subsequently quantified using Nutil, as described above. Manual counting was performed for each section throughout the calbindin stained anterior cingulate cortex (sections s103 – s175 from mouse 10), the parvalbumin stained entorhinal cortex (s019 – s044 from rat 25205), and for one whole hemisphere from each of the stains (s151 from mouse10 and s028 from rat 25205). The regions of interest were defined by the QuickNII atlas maps for the evaluated sections and were thus identical for the quantification of manual and ilastik segmentations.

Reliability of segmentations

We assessed both intra- and interrater reliability by segmenting material from one of the series (parvalbumin stained sections from rat 25205) several times. Intrarater reliability was assessed by one researcher segmenting the same material three times. The first and second segmentation was performed within the same week, while the last one was done six weeks later.

In a pilot study of interrater reliability, we considered the reliability of segmentations obtained by three researchers using the same material, but without clear guidelines for what to consider a labelled cell. We observed, both qualitatively and quantitatively, that results varied considerably between the three researchers. We therefore set out to do a more systematic assessment of interrater reliability, with clear instructions on how to perform the segmentation with ilastik. One researcher performed an initial segmentation and wrote instructions for its replication. The same materials and instructions were then presented to five other researchers for segmentation. The instructions gave a description of objects considered to be cells and example images of segmentation results. In addition, information was given on how to train the ilastik classifier, with instruction to not label pixels in oversaturated areas (specifically, the cerebellum and reticular nucleus of thalamus). Researchers were also given access to the segmentations overlaid on the original (high-resolution) images via an online viewer system, and were instructed to use these actively to verify similarity of segmentations. The full instructions as given to the researchers is included in the section below. Each researcher then ran their classifier on the full set of images from rat 25205. Objects in the resulting segmented images were quantified using Nutil quantifier, as described above.

Documentation for segmentation of parvalbumin cells in rat 25205

Instructions provided to researchers:

Goal. The goal of this exercise is to reproduce a segmentation (“target segmentation”) made by one researcher as faithfully as possible. The original segmentation was made with the aim of extracting parvalbumin positive cell bodies from DAB-stained images. The cells are generally easy to recognize, but there are variations across the material that makes some profiles more difficult to determine. In the more difficult cases, researchers might have different opinions on what should be considered a cell. I have aimed to provide the sufficient and necessary documentation with respect to what I have considered a cell when segmenting this material. It is important that you consider the provided examples and attempt to replicate this, regardless of whether you agree with the definitions and segmentations made here.

Training images. The training material includes very fifth section throughout the series, starting with s003 and ending with s053. Images are 15 % of original tiff resolution (with original tiffs being 50 % of original CZI file).

Cell criteria. A combined evaluation of the following criteria was used to decide whether an object is a cell or not:

- clearly distinguished from the background
- round, ovoid, triangular, or multipolar in shape
- medium, darkly, or intensely stained
- size substantial enough to support that the object is a cell and not axonal or dendritic fragments

See “Examples of manually annotated cells” below for examples.

Training in ilastik. Certain areas are so heavily labelled or contains so much stained neuropil that they should be excluded from the brain-wide analysis so as not to compromise the quality of the remaining classifier. For this reason, do not segment pixels in the cerebellum or reticular nucleus of the thalamus. Before starting, see the examples of manual annotations and target segmentations in the end of this document. High-resolution images with target segmentations overlaid are available via the Navigator3 system, and can be used as a reference during segmentation.

1) Select all features
2) Create two labels: cells and background
3) Use the thinnest brush stroke (1 px)
4) Label some clear examples of cells, and label directly adjacent pixels as background.

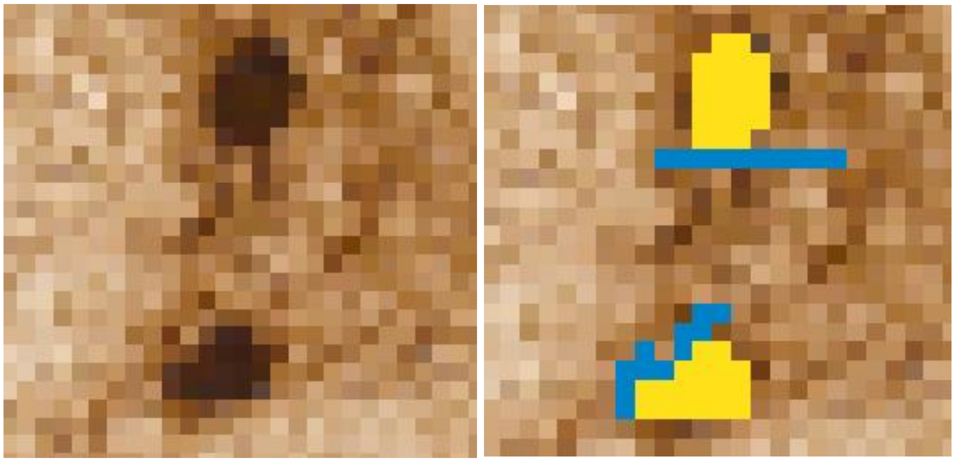

Step 2 illustration

5) Label some cells that are closely placed, and label the area between them as background
6) Place some background labels in appropriate areas, especially in regions with a lot of stained neuropil and around ventricles where staining is strong but not representing cells.

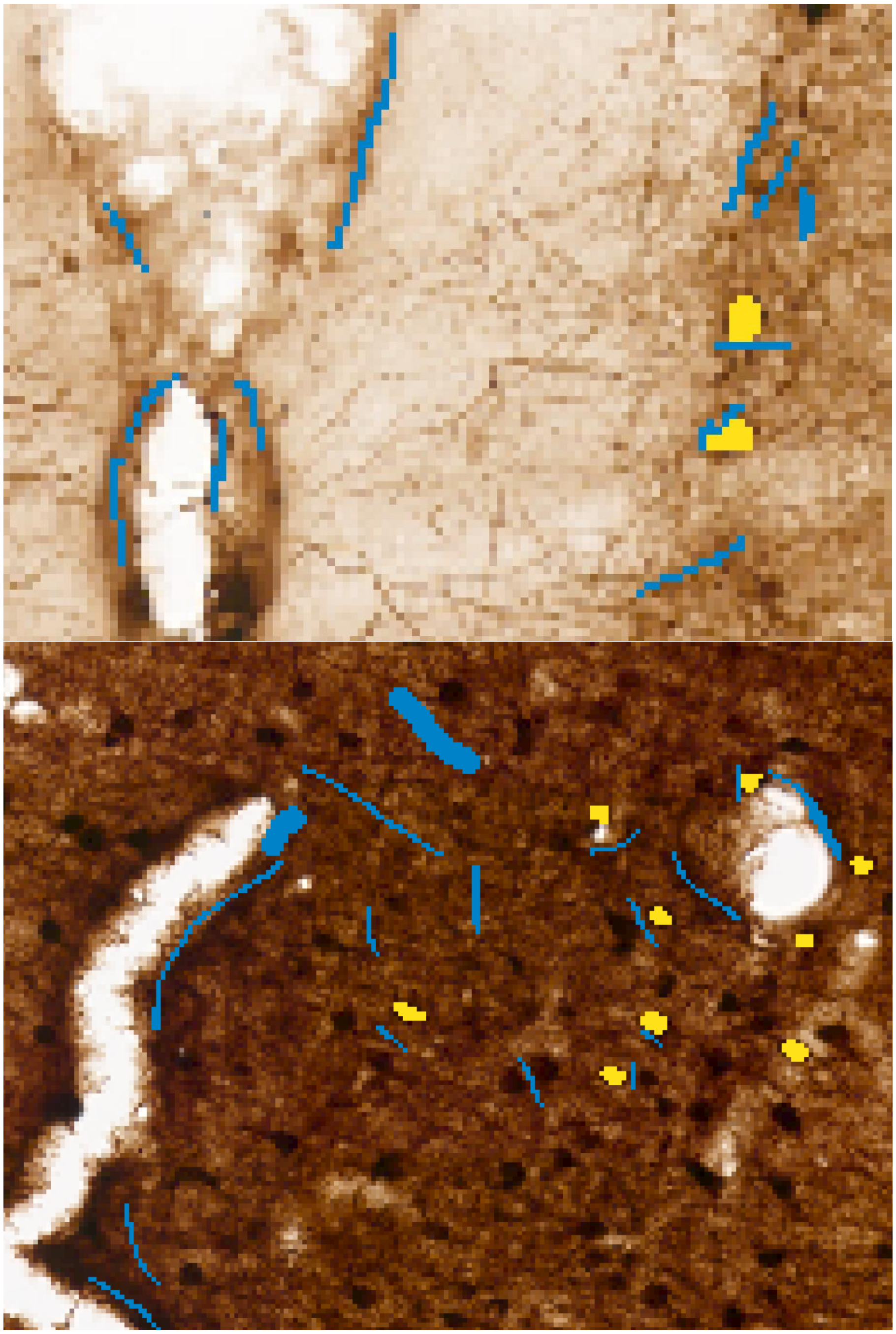

Step 3-4 illustration

7) Make sure to do steps 3-5 for areas of different intensities and across anatomical regions (cortex, hippocampus, striatum) and training sections. Use a bigger (5 or 7 px) brush to label more extensive areas of background.
8) Use live update and toggle the uncertainty filter to identify objects for which the classifier needs further input
9) Label cells for which the classifier is uncertain until the uncertainty is restricted to haloes surrounding cells, i.e. until there is little uncertainty in the center of the objects. Make sure to label areas of background at the same time.

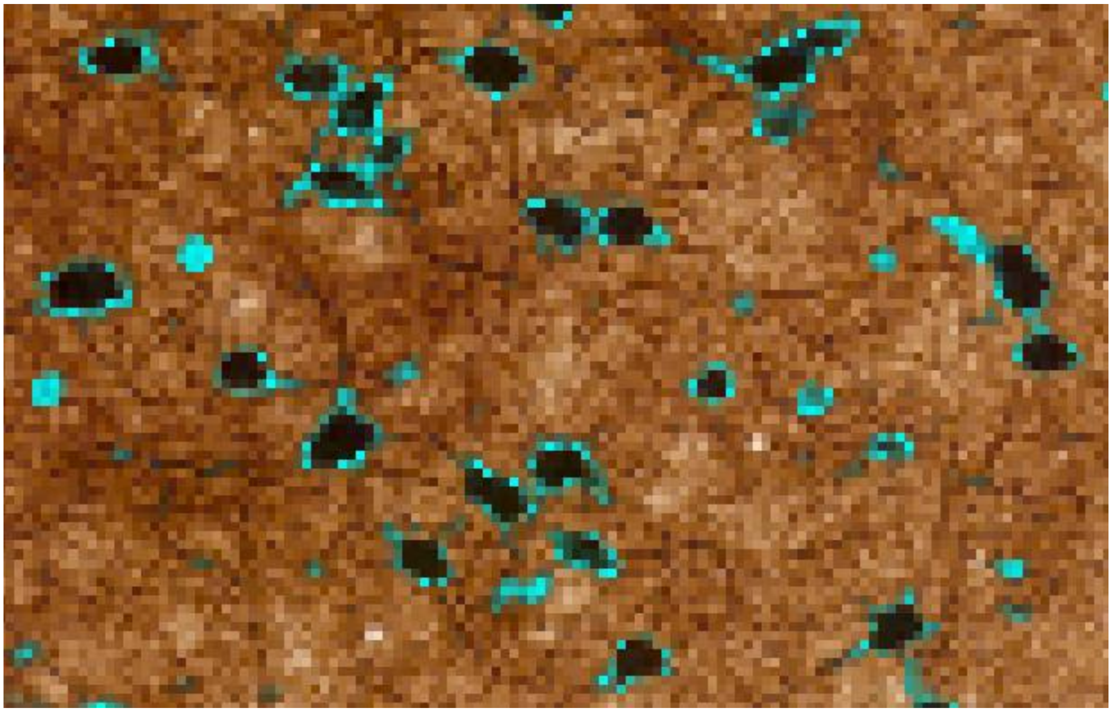
Uncertainty filter applied. Cells are recognized with confidence, despite uncertainty at their edges.

10) Repeat step 8 across all training images, and make sure to visit representative areas (including cortex, striatum, hippocampus, globus pallidus, basal forebrain, olfactory bulb).
11) See target segmentation images in Navigator to evaluate whether results are satisfactory. Go through all training images with reference to the segmented training images. If you have a lot of undesired pixels segmented as cells, label more background pixels. Note that a pixel size filter of 4 pixels will be applied to the segmentations, so fragments smaller than this will not affect the results.

Examples of manually annotated cells

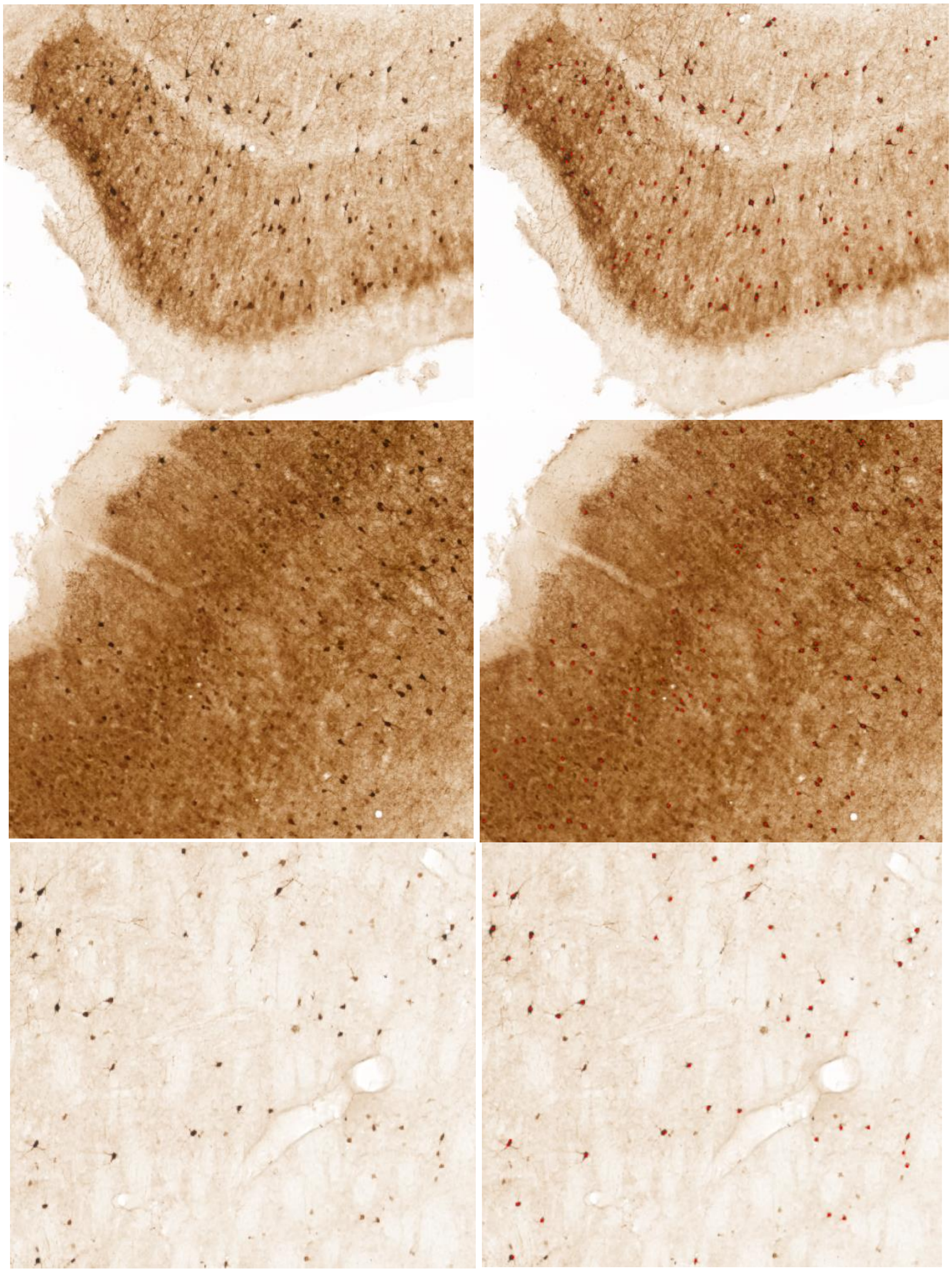

Examples images (left) and the same images with manually annotated cells. The examples are from medial entorhinal cortex (upper), neocortex (middle) and striatum (lower) from s028 of the training images. Very lightly stained objects, or objects that do not have a clear cell shaped outline easily distinguished from background staining, are here not considered to represent cell profiles.

Examples of target segmentation results

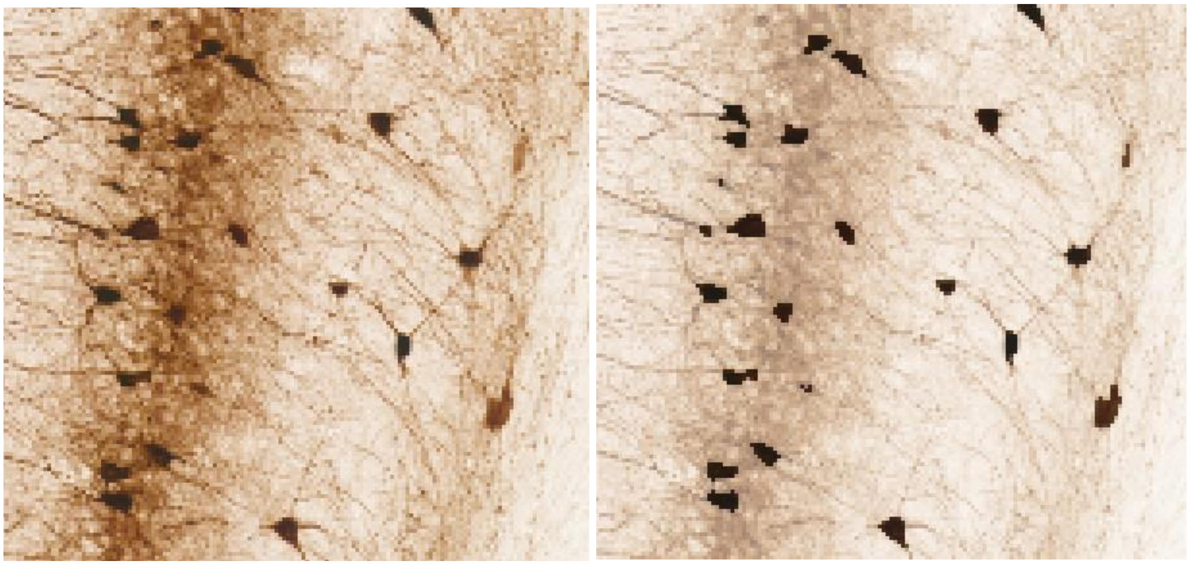

Hippocampus, s014

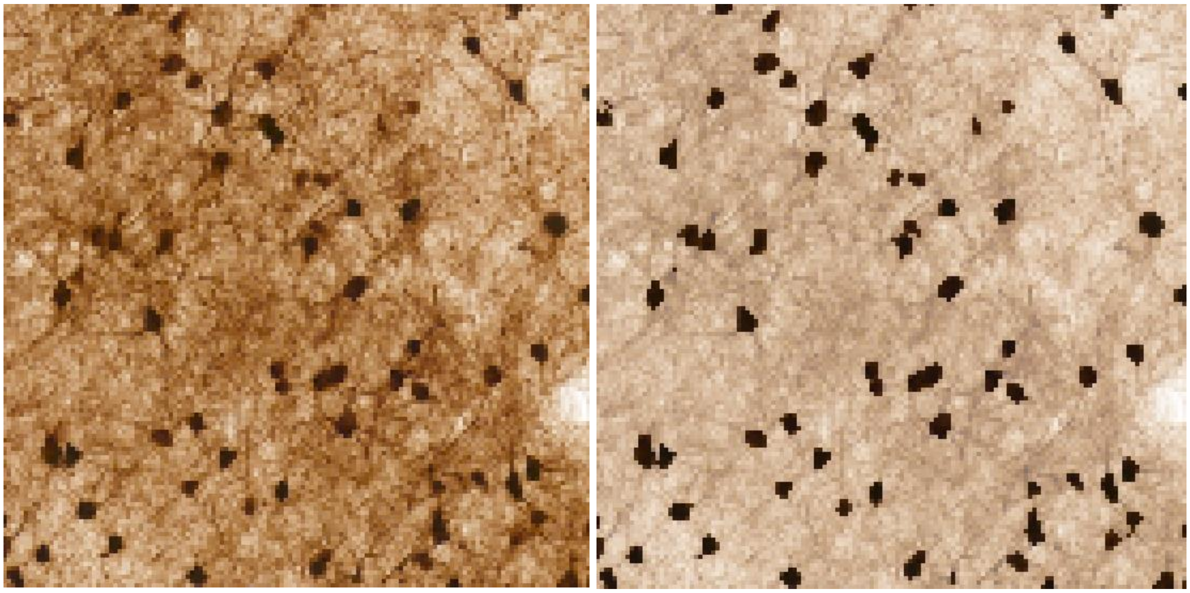

Cortex, s014

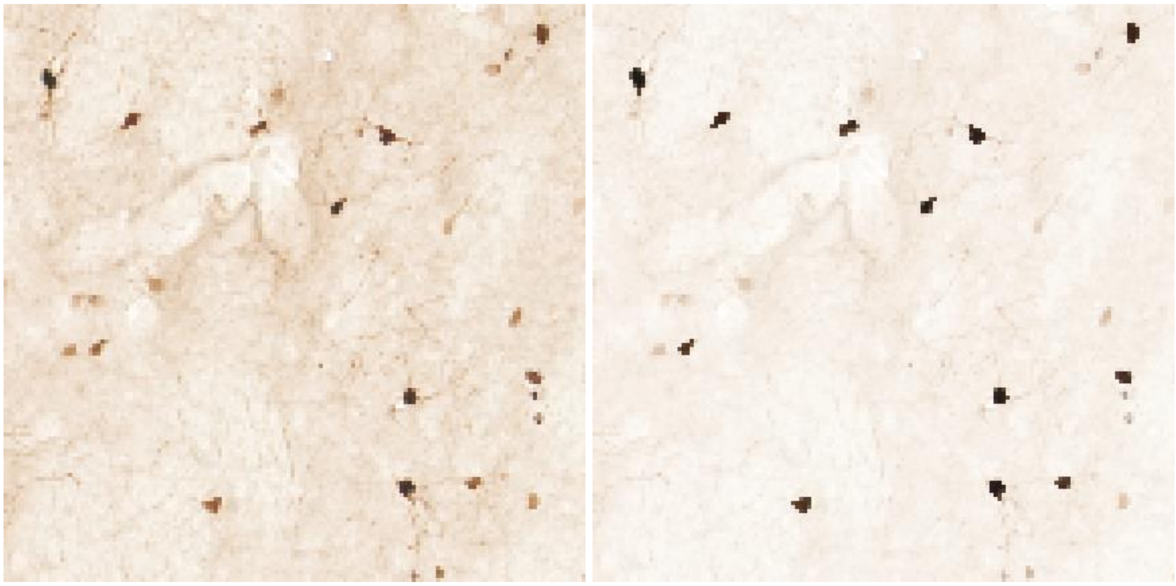

Caudoputamen, s025

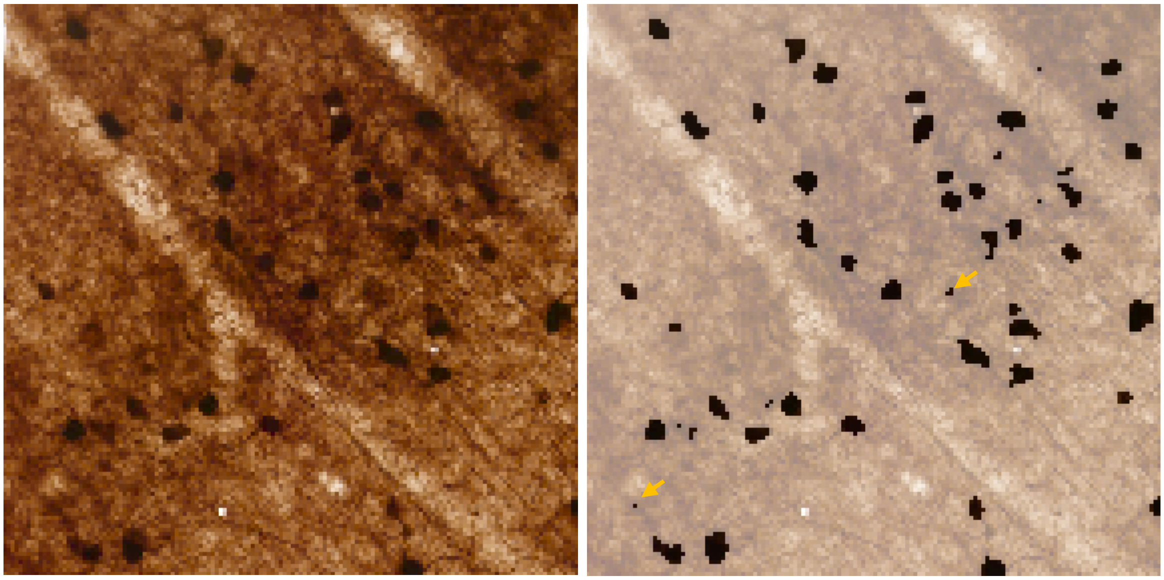

Cortex, s025. Yellow arrows point to examples of fragments that will be filtered out later based on size and thus will not affect count results.

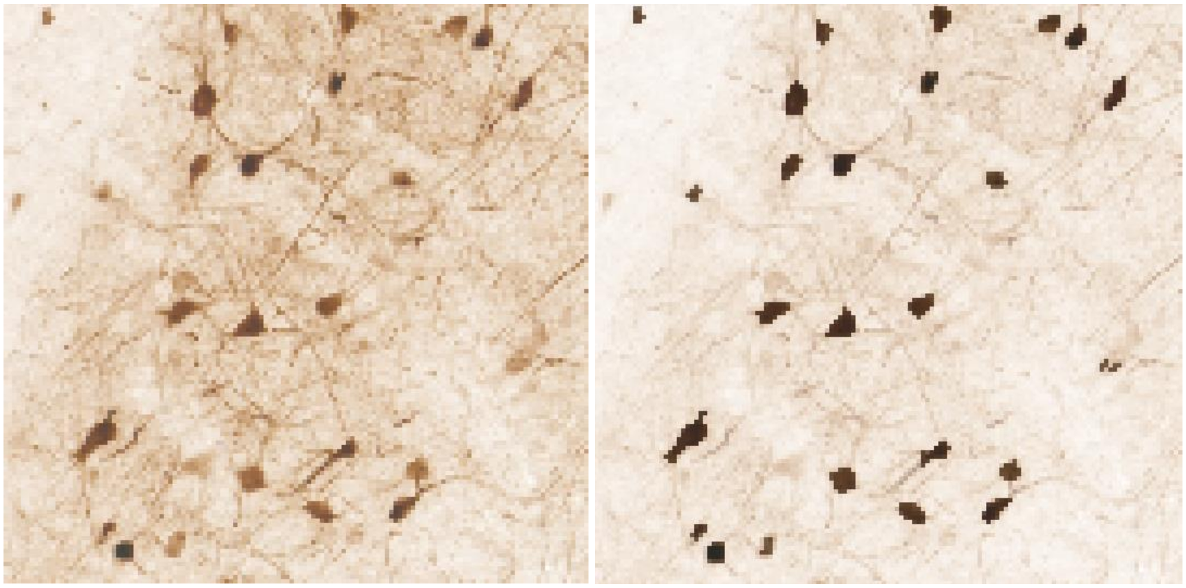

Globus pallidus, s038

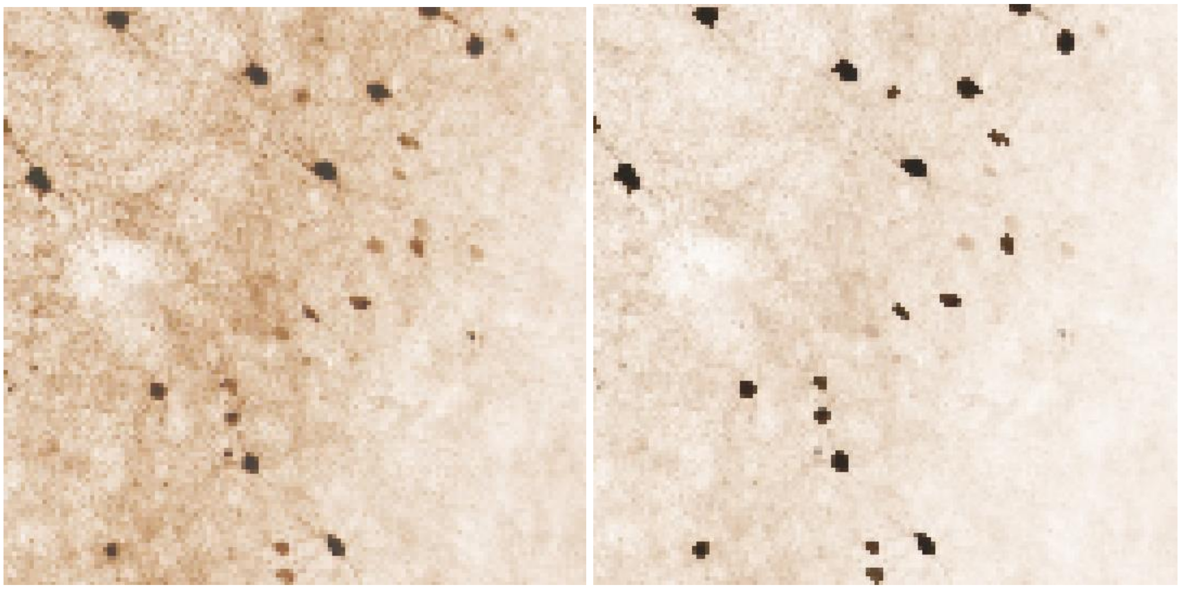

Cortex, s038

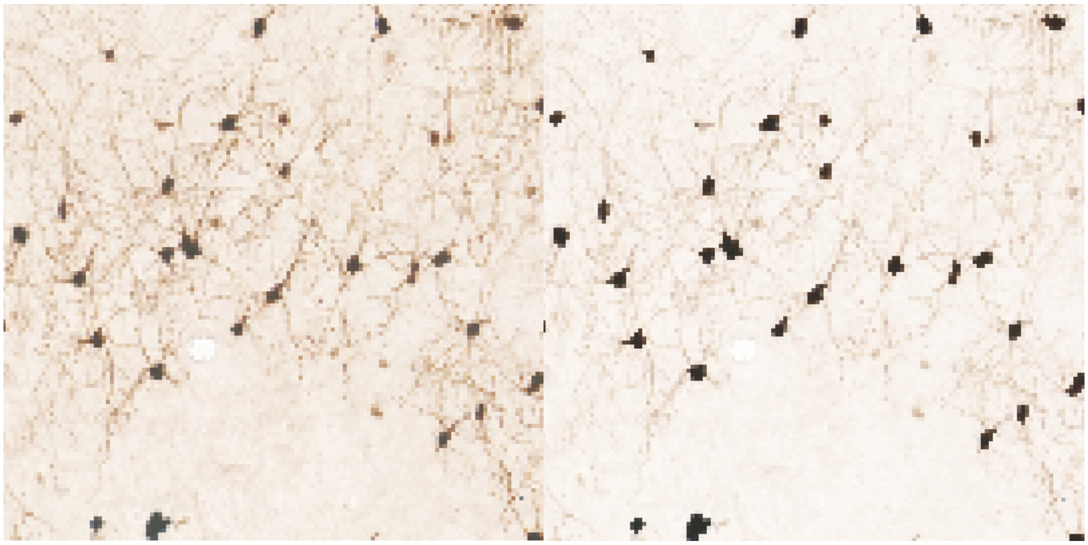

Olfactory bulb, s038

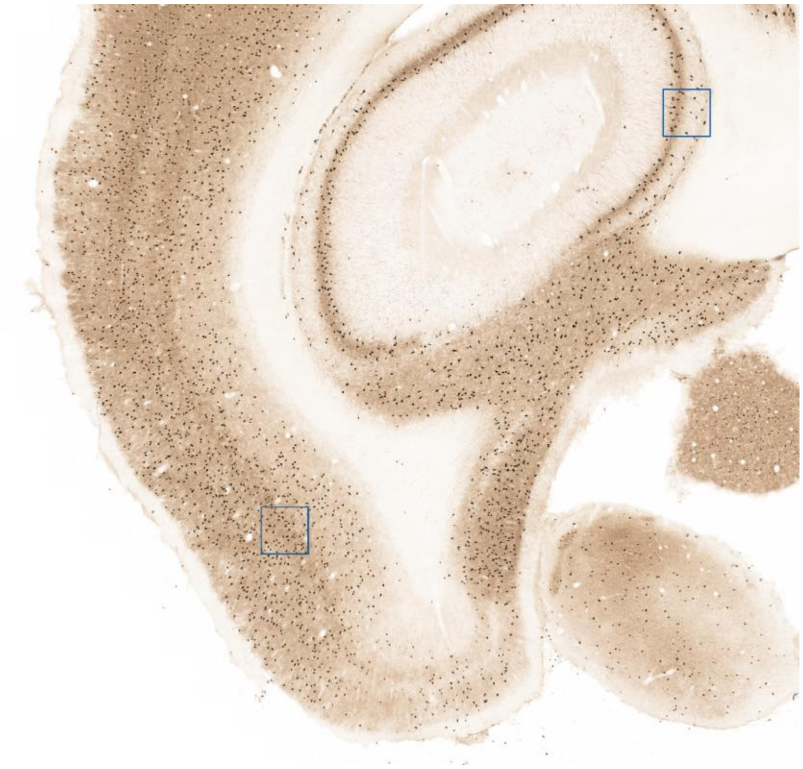

Hippocampus and cortex parts shown in examples above, s014

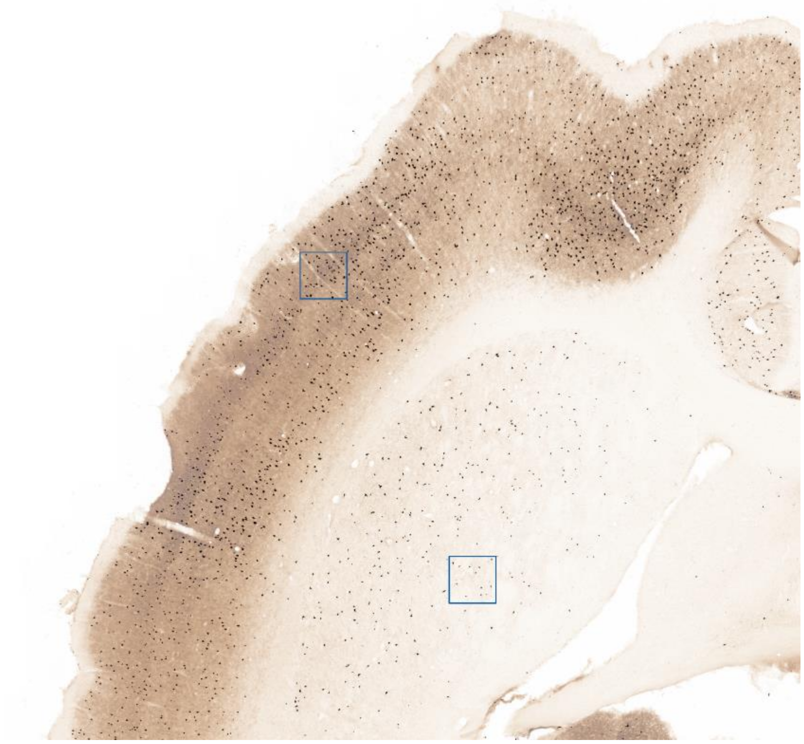

Caudoputamen and cortex parts shown in examples above, s025

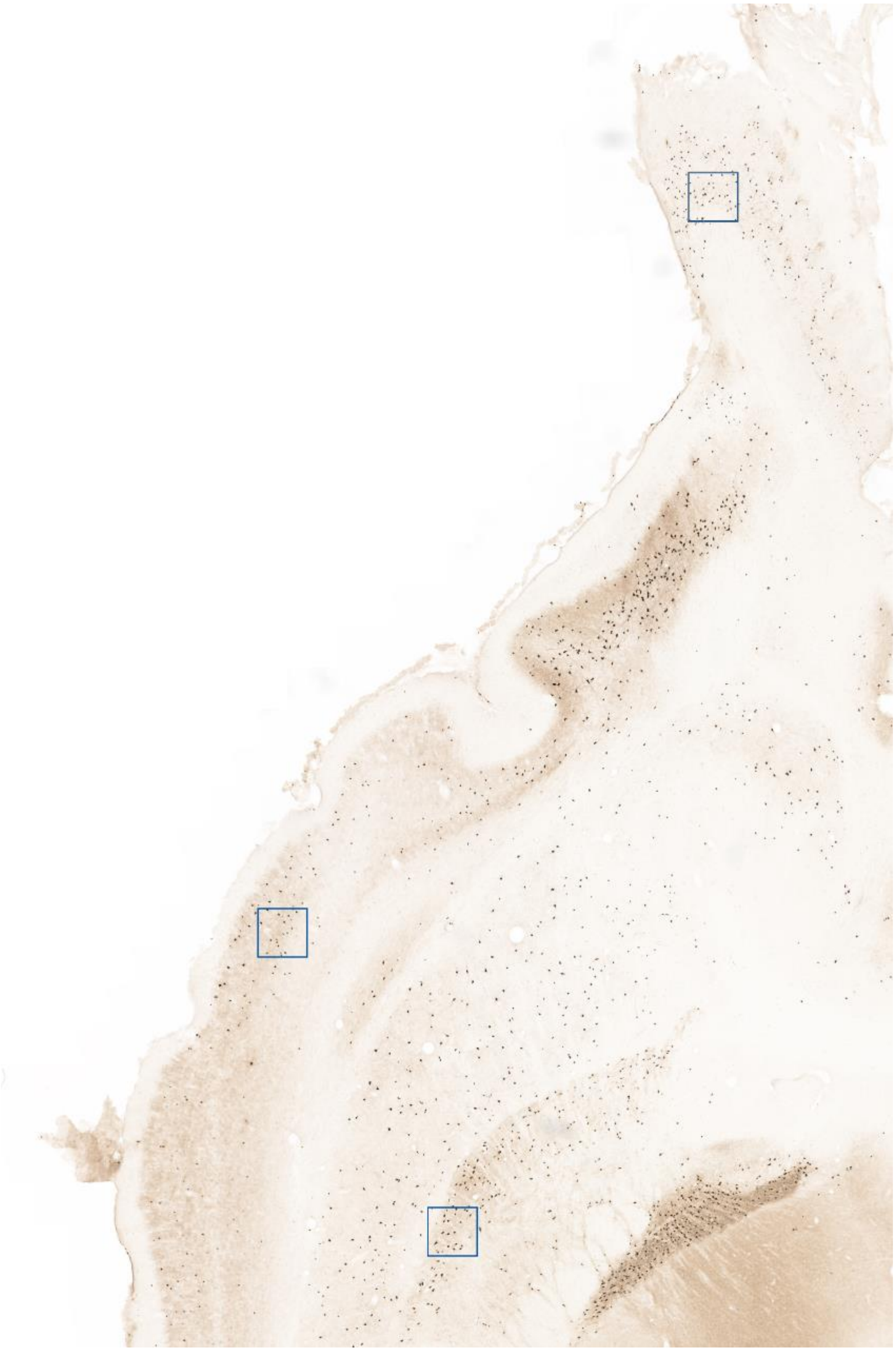

Globus pallidus, cortex, and olfactory bulb parts shown in examples above, s038

QUANTIFICATION AND STATISTICAL ANALYSIS

Quantitative data throughout this paper are reported as mean ± SEM. Summary statistics were extracted using Microsoft Excel (RRID:SCR_016137). Exact values of n, representing the number of animals, are given in the result section.

Supplemental references

Aika, Y., Ren, J.Q., Kosaka, K., and Kosaka, T. (1994). Quantitative analysis of GABA-like-immunoreactive and parvalbumin-containing neurons in the CA1 region of the rat hippocampus using a stereological method, the disector. Exp. Brain Res. *99*, 267–276.

Barinka, F., Salaj, M., Rybář, J., Krajčovičová, E., Kubová, H., and Druga, R. (2012). Calretinin, parvalbumin and calbindin immunoreactive interneurons in perirhinal cortex and temporal area Te3V of the rat brain: Qualitative and quantitative analyses. Brain Res. *1436*, 68–80.

Bjerke, I., Puchades, M., Bjaalie, J., and Leergaard, T. (2019). Database of quantitative cellular and subcellular morphological properties from rat and mouse basal ganglia (Human Brain Project Neuroinformatics Platform).

Kaalund, S.S., Riise, J., Broberg, B. V., Fabricius, K., Karlsen, A.S., Secher, T., Plath, N., and Pakkenberg, B. (2013). Differential expression of parvalbumin in neonatal phencyclidine-treated rats and socially isolated rats. J. Neurochem. *124*, 548–557.

Kägi, U., Berchtold, M.W., and Heizmann, C.W. (1987) Ca^{2+}-binding parvalbumin in rat testis. Characterization, localization, and expression during development. J. Biol. Chem., *262*(15), 7314-7320.

Megahed, T., Hattiangady, B., Shuai, B., and Shetty, A.K. (2015). Parvalbumin and neuropeptide Y expressing hippocampal GABA-ergic inhibitory interneuron numbers decline in a model of Gulf War illness. Front. Cell. Neurosci. *8*, 1–12.

Moene, I.A., Subramaniam, S., Darin, D., Leergaard, T.B., and Bjaalie, J.G. (2007). Toward a workbench for rodent brain image data: Systems architecture and design. Neuroinformatics.

Pinol, M.R., Kägi, U., Heizmann, C.W., Vogel, B., Sequier, J.M., Haas, W., Hunziker. (1990) Poly- and monoclonal antibodies against recombinant rat brain calbindin D-28 K were produced to map its selective distribution in the central nervous system. J. Neurochem. *54*(6), 1827-1833.

Sanchez-Mejias, E., Nuñez-Diaz, C., Sanchez-Varo, R., Gomez-Arboledas, A., Garcia-Leon, J.A., Fernandez-Valenzuela, J.J., Mejias-Ortega, M., Trujillo-Estrada, L., Baglietto-Vargas, D., Moreno-Gonzalez, I., et al. (2020). Distinct disease-sensitive GABAergic neurons in the perirhinal cortex of Alzheimer's mice and patients. Brain Pathol. *30*, 345–363.

Shiraki, A., Tanaka, T., Watanabe, Y., Saito, F., Akahori, Y., Imatanaka, N., Yoshida, T., and Shibutani, M. (2016). Immunohistochemistry of aberrant neuronal development induced by 6-propyl-2-thiouracil in rats. Toxicol. Lett. *261*, 59–71.

Taube, J. (2007). The head direction signal: Origins and sensory-motor integration. Annu. Rev. Neurosci. *30*, 181–207.

Taube, J.S., Muller, R.U., and Ranck, J.B. (1990). Head-direction cells recorded from the postsubiculum in freely moving rats. II. Effects of environmental manipulations. J. Neurosci. *10*, 436–447.

Trujillo-Estrada, L., Dávila, J.C., Sánchez-Mejias, E., Sánchez-Varo, R., Gomez-Arboledas, A., Vizuete, M., Vitorica, J., and Gutiérrez, A. (2014). Early neuronal loss and axonal/presynaptic damage is associated with accelerated amyloid-β accumulation in AβPP/PS1 Alzheimer's disease mice subiculum. J. Alzheimer's Dis. *42*, 521–541.

Wang, C.Z., Yang, S.F., Xia, Y., and Johnson, K.M. (2008). Postnatal phencyclidine administration selectively reduces adult cortical parvalbumin-containing interneurons. Neuropsychopharmacology *33*, 2442–2455.
